# Lipidomics reveals the pro-viral roles of ceramides during fish nodavirus infection

**DOI:** 10.1128/jvi.01991-25

**Published:** 2026-02-23

**Authors:** Ya Zhang, Lin Liu, Long Lin, Yin Zhao, Kaitao Xi, Xixi Guo, Qiwei Qin, Xiaohong Huang, Youhua Huang

**Affiliations:** 1 College of Marine Sciences, South China Agricultural University 12526, Guangzhou, China; 2 Nansha-South China Agricultural University Fishery Research Institute, Guangzhou, China; 3 Southern Marine Science and Engineering Guangdong Laboratory 590852 , Zhuhai, China; University of Michigan Medical School, Ann Arbor, Michigan, USA

**Keywords:** RGNNV, lipid metabolism, ceramides, pro-viral, autophagy

## Abstract

Red-spotted grouper nervous necrosis virus (RGNNV) is one of the most lethal viral pathogens with high mortality in the fry and juvenile stage of marine fish. Our previous studies demonstrated that RGNNV infection induced the remodeling of intracellular membrane and exploited cellular fatty acid synthesis for infection. However, the roles of lipid metabolism during infection still remained largely uncertain. Here, the global lipidomic profiles of RGNNV in grouper cells were analyzed, and the crucial roles of ceramides during viral infection were investigated. RGNNV significantly altered lipid homeostasis *in vitro*. Of note, almost all the detected ceramides were elevated in RGNNV-infected cells. Consistently, RGNNV infection induced a significant increase in ceramides accumulation and the mRNA expression levels of ceramide synthesis–related genes. Interestingly, virus-induced ceramides were colocalized with RGNNV coat protein (CP), but not RNA-dependent RNA-polymerase (RdRp). Furthermore, ectopic expression of CP alone also induced a significant increase in the levels of ceramide species, suggesting that RGNNV manipulated the ceramide metabolism partially via CP protein. Furthermore, disruption of ceramide synthesis pathways using pharmacological inhibitors or knockdown technology markedly suppressed RGNNV infection, and the inhibitory effects were rescued by the exogenous C16-ceramide (d18:1/16:0). In addition, C16-ceramide enhanced RGNNV-induced autophagy and reversed the antiviral action of an autophagy inhibitor chloroquine upon RGNNV infection, suggesting that ceramides might promote RGNNV replication via autophagy. Together, our findings firstly demonstrated that RGNNV altered sphingolipid metabolism and ceramide flux was one of pro-viral mediators for RGNNV replication, providing potential approaches for preventing fish nodaviral infection.

**IMPORTANCE:**

Ceramides, as central intermediates in sphingolipid metabolism, are involved in regulating cell viability, differentiation, cycle autophagy, and immune response. Viruses can regulate ceramide synthesis by driving different ceramide synthesis pathways, resulting in differential utilization of ceramides during virus infection. In this study, we demonstrated that RGNNV infection and CP overexpression altered lipid homeostasis, especially by inducing sphingolipid metabolism. Three major ceramide synthesis pathways*—de novo* biosynthesis, salvage, and sphingomyelin degradation—were all required for RGNNV infection. Moreover, the addition of C16-ceramide (d18:1/16:0) significantly promoted RGNNV replication via increasing RGNNV-induced autophagy. Our findings not only contribute greatly to understanding the mechanism underlying fish nodavirus pathogenesis, but also shed new light on the potential of ceramides as an ideal candidate for prevention and treatment for viral nervous necrosis (VNN).

## INTRODUCTION

Nodavirus is a non-enveloped positive-strand RNA [(+) RNA] virus belonging to the family *Nodaviridae*. As single genome (+) RNA viruses, nodaviruses, flaviviruses, and bromoviruses have been demonstrated to induce the rearrangement of organelle membrane, supporting the replication of viral genomes (1, 2). For example, flock house virus (FHV), a member in genus *Alphanodavirus* in the family *Nodaviridae*, induced the formation of invaginations in the outer mitochondrial membrane and replicated its RNA on mitochondrial membranes during viral replication and assembly (3, 4). Moreover, these (+) RNA viruses extensively rewired lipid metabolism and remodeled cellular membranes to form viral replication compartments (5–7). The membrane remodeling occurring in dengue virus (DENV)-infected cells was directly linked to a unique lipid repertoire (6). On the other hand, viruses could utilize lipids to facilitate viral replication in multiple stages of the viral cycle. For example, glycerophospholipids (GPs) were required for the effective replication of FHV *in vitro* (8).

Nervous necrosis virus (NNV), a member in genus *Betanodavirus* in the family *Nodaviridae*, causes viral encephalopathy and retinopathy (VER) or viral nervous necrosis (VNN) disease in aquaculture. Up to now, a number of NNV isolates have been identified from more than 120 fish species (9, 10). Red-spotted grouper nervous necrosis virus (RGNNV), an important etiological agent of viral diseases in marine fish, always causes up to 100% mortality in larval and juvenile fish in the aquaculture industry (10). RGNNV genome consists of two single-stranded positive-sense RNAs, which encode RNA-dependent RNA-polymerase (RdRp) and capsid protein (CP), respectively (11). The typical pathological feature of NNV infection was the dramatic cytoplasmic vacuoles evoked by virus infection *in vitro* (12) and *in vivo* (13). Recent studies revealed that RGNNV induced autophagy (14), which participated in lysosomal vacuolation-mediated cell death during virus infection (12). Single-cell RNA-seq of RGNNV-infected midbrain cells showed that key genes and pathways involved in cell cytoplasmic vacuoles and autophagy were significantly enriched (15). Electron microscopy observation indicated that in addition to swollen lysosomes, monolayer-membrane structures (viral replication compartments), which contained numerous virus particles, were observed in RGNNV-infected cells, suggesting that RGNNV infection induced the remodeling of intracellular membrane (12). Recent studies emphasized the important roles of host metabolism in RGNNV infection, such as glutamine metabolism and fatty acid synthesis (16, 17). However, the potential mechanisms by which RGNNV regulated the lipid metabolic network of host cells, and the roles of lipid metabolites in virus infection, still remained uncertain.

Sphingolipids (SPs) are a kind of important bioactive lipids in eukaryotic cell membranes and mainly include five members: ceramide, sphingomyelin, ceramide-1-phosphate, sphingosine, and sphingosine 1-phosphate (18). Ceramides, as central intermediates in sphingolipid metabolism, are involved in regulating cell proliferation, differentiation, migration, apoptosis, autophagy, and inflammation (19). Notably, several studies have demonstrated that ceramides exerted different roles upon various virus infections (20, 21). For example, ceramide production via the *de novo* and salvage pathways was required for replication of West Nile virus (WNV) (22). Ceramides redistributed to the Zika virus (ZIKV) replication sites, and increasing ceramide levels by various pathways sensitized cells to ZIKV infection (23). In contrast, influenza A virus (IAV) infection triggered ceramides accumulation through *de novo* biosynthesis pathway, and the addition of exogenous C6-ceramide suppressed IAV replication (24). Moreover, ceramide metabolism was speculated to be a potential target against viral infections (21, 25, 26). Therefore, it is urgent to investigate the pro-viral or antiviral actions of ceramides in the replication of aquatic vertebrate viruses.

In the present study, global lipidomic profiles of RGNNV-infected cells and CP-overexpressing cells were investigated. Using pharmacological inhibitors, knockdown of enzymes involved in ceramide synthesis, as well as the addition of exogenous ceramide, we elucidated the actions of ceramides during RGNNV infection. Our results will provide novel insights into the changes of lipid metabolites and the function of ceramide metabolism in fish virus infection.

## RESULTS

### Lipid metabolic profiles of grouper cells in response to RGNNV infection

**Fig 1:**
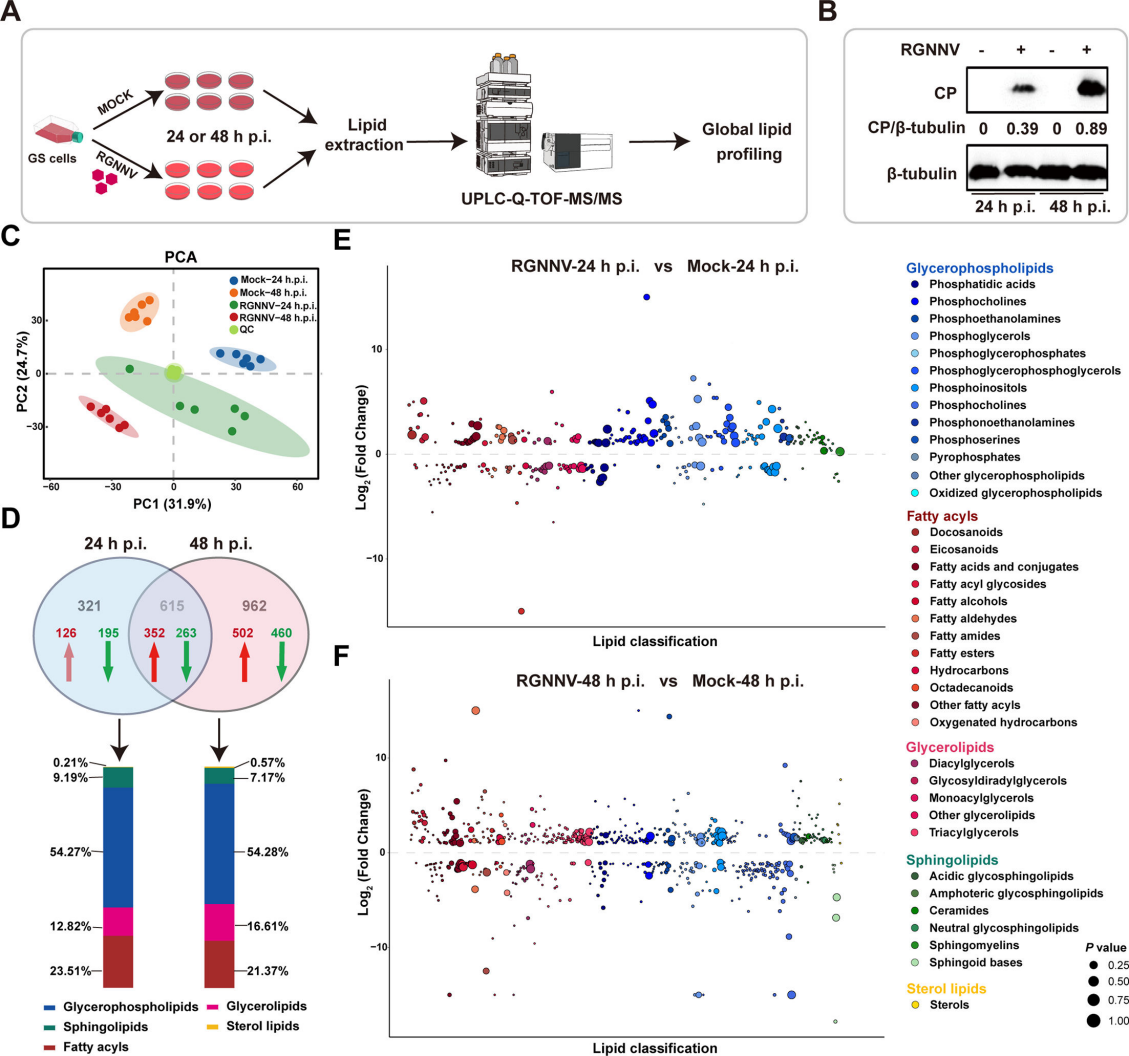
Global lipidomics of RGNNV-infected GS cells. (**A**) Overview and workflow of the global lipidomics in mock- or RGNNV-infected cells. GS cells (*n* = 6) were infected with RGNNV (MOI = 2) for 24 or 48 h, and then mock- (24 or 48 h p.i.) and RGNNV-infected cells (24 or 48 h p.i.) were harvested for lipid metabolomics. (**B**) The protein level of CP in mock- or RGNNV-infected cells. GS cells infected with RGNNV were collected at 24 or 48 h p.i. to determine the CP protein expression by western blotting assay. (**C**) PCA of the lipidomics data set. (**D**) Venn diagram for comparison of differential lipid metabolites between RGNNV-infected groups. Green and red arrows represented the downregulated metabolites and upregulated metabolites, respectively. (**E, F**) Bubble plots of log2 (Fold change) in abundance of lipid species in RGNNV-infected cells relative to mock at 24 h p.i. (**E**) and 48 h p.i. (**F**). Bubble size represents *P* value from *t*-test.

Our previous studies showed that RGNNV infection induced remodeling of intracellular membrane and exploited cellular fatty acid synthesis in grouper spleen (GS) cells for virus infection (12, 17). To further investigate whether cellular lipid metabolism was involved in RGNNV infection, a global lipidomic profile of RGNNV-infected cells was carried out. The lipid extraction of GS cells (*n* = 6) at 24 and 48 h post-infection (p.i.) was analyzed using UPLC-Q-TOF-MS/MS (Fig. 1A). The protein level of CP gradually increased with infection time, indicating that GS cells were successfully infected with RGNNV (Fig. 1B). Principal component analysis (PCA) confirmed that four groups in different infection status (mock or RGNNV) and infection time points (24 or 48 h p.i.) were gathered together, respectively (Fig. 1C), indicating that RGNNV infection accounted for changes in lipid metabolism. We identified a total of 1,898 lipid metabolites, spanning SPs, GPs, glycerolipids (GLs), fatty acyls (FAs), and sterol lipids (STs) (Table S1). Of these, 615 metabolites showed differential abundance at both two infection time points, while 321 and 962 metabolites were significantly altered only at 24 and 48 h p.i., respectively (Fig. 1D).

Next, we examined how RGNNV infection altered host lipid composition at the subclass at 24 and 48 h p.i. (Fig. 1E and F). Trends among GP subclasses were similar: many phosphatidic acids (PA), phosphoglycerols (PG), phosphoinositols (PI), phosphoserines (PS), phosphoethanolamines (PE), and phosphocholines (PC) were enriched at 24 and 48 h p.i., especially PC and PI species (Table S1; Fig. S1A through H). As the main identified GLs, triacylglycerols (TAG) were stored within lipid droplets, which provided energy substrates to increase metabolism during virus infection (27). In line with evidence that lipid droplets were formed during RGNNV infection (17), most of TAG and diacylglycerols (DAG) increased at 48 h p.i. (Table S1; Fig. S1I and J). As shown in Table S1 and Fig. S2A through L, the levels of many FAs were elevated in RGNNV-infected cells, which were similar to our previous study that RGNNV induced and exploited cellular fatty acid synthesis for virus infection (17). In addition, most cholesteryl esters were differently increased during RGNNV infection (Fig. S2M), consistent with the trends in ZIKV-infected cells (23).

### RGNNV infection enriched sphingolipids (SPs) *in vitro*

**Fig 2:**
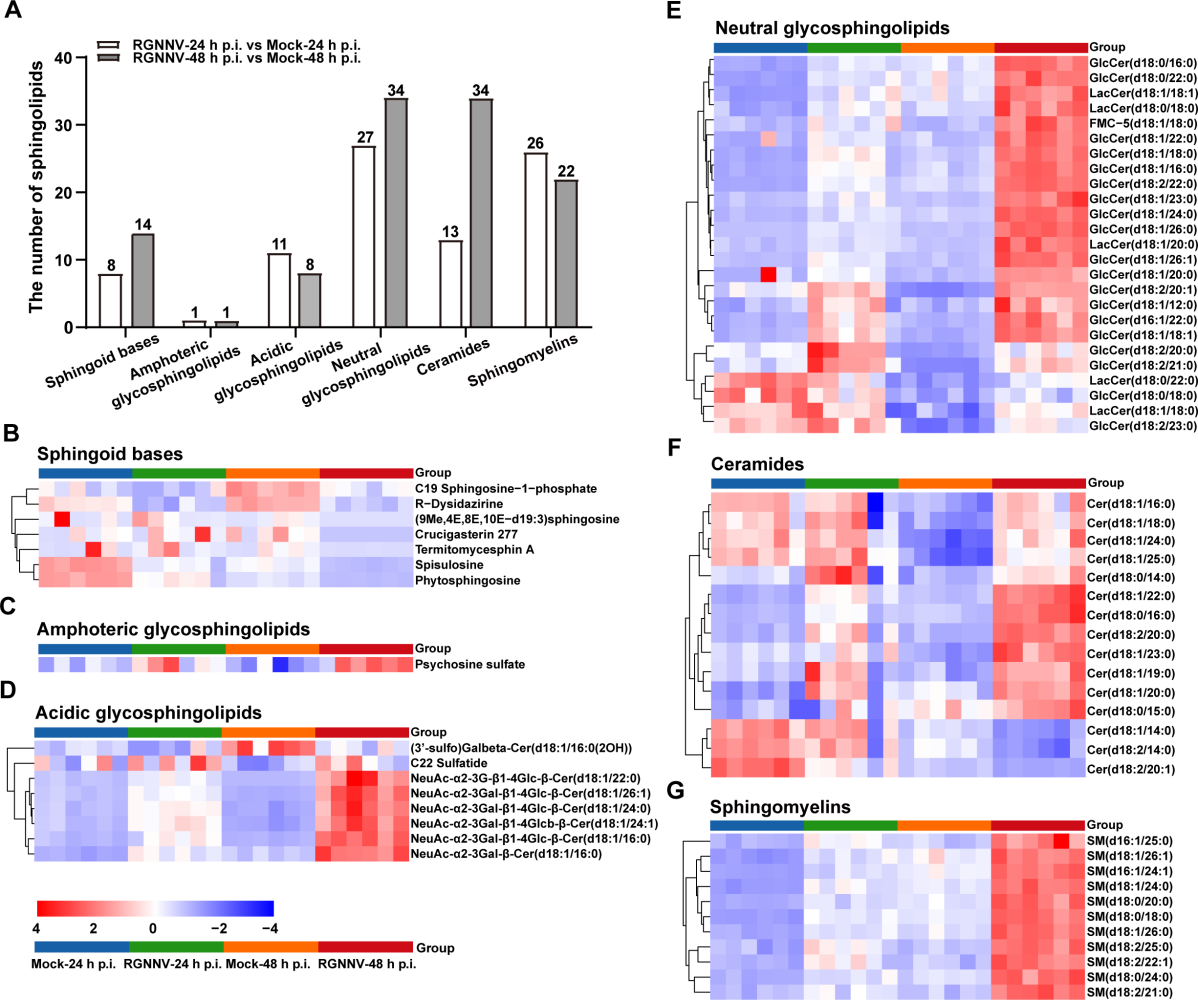
RGNNV infection altered the SP levels in GS cells. (**A**) The number of altered lipid metabolites in SP subclasses in RGNNV-infected cells. (**B–G**) The heatmap of representative SPs. Except for the identified sphingoid bases (**B**), most amphoteric glycosphingolipids (**C**), acid glycosphingolipids (**D**), neutral glycosphingolipids (**E**), ceramides (**F**), and sphingomyelins (**G**) were elevated in RGNNV-infected cells. The heatmap displayed the expression patterns of differential SPs according to the criteria of |FC| > 2 or < 1 and *P* < 0.05. Data were normalized by z-score and subjected to hierarchical clustering. The color gradient from blue (negative z-score, low expression) to red (positive z-score, high expression) indicated the degree of expression deviation.

Upon RGNNV infection at 24 and 48 h p.i., a total of 86 and 113 species of SPs were significantly altered, respectively (Table S1). As shown in Fig. 2A, these changed SP species were classified into six subclasses: sphingoid bases (SB), ceramides (Cer), sphingomyelins (SM), amphoteric glycosphingolipids, neutral glycosphingolipids (NGSL), and acidic glycosphingolipids (AGSL). Interestingly, except for SB species (Fig. 2B), the abundance of almost all amphoteric glycosphingolipids (Fig. 2C), AGSL (Fig. 2D), NGSL (Fig. 2E), Cer (Fig. 2F), and SM (Fig. 2G) detected in RGNNV-infected cells was increased at the late stage of virus infection. For example, among the identified 34 ceramides in RGNNV-infected cells at 48 h p.i., 28 ceramides were increased, including Cer(d18:1/22:0), Cer(d18:0/16:0), Cer(d18:2/20:0), and Cer(d18:1/23:0) (Table S1; Fig. 2F). Thus, these results indicated that RGNNV infection disturbed lipid homeostasis and enriched SPs *in vitro*.

### RGNNV infection triggered the ceramides accumulation

**Fig 3:**
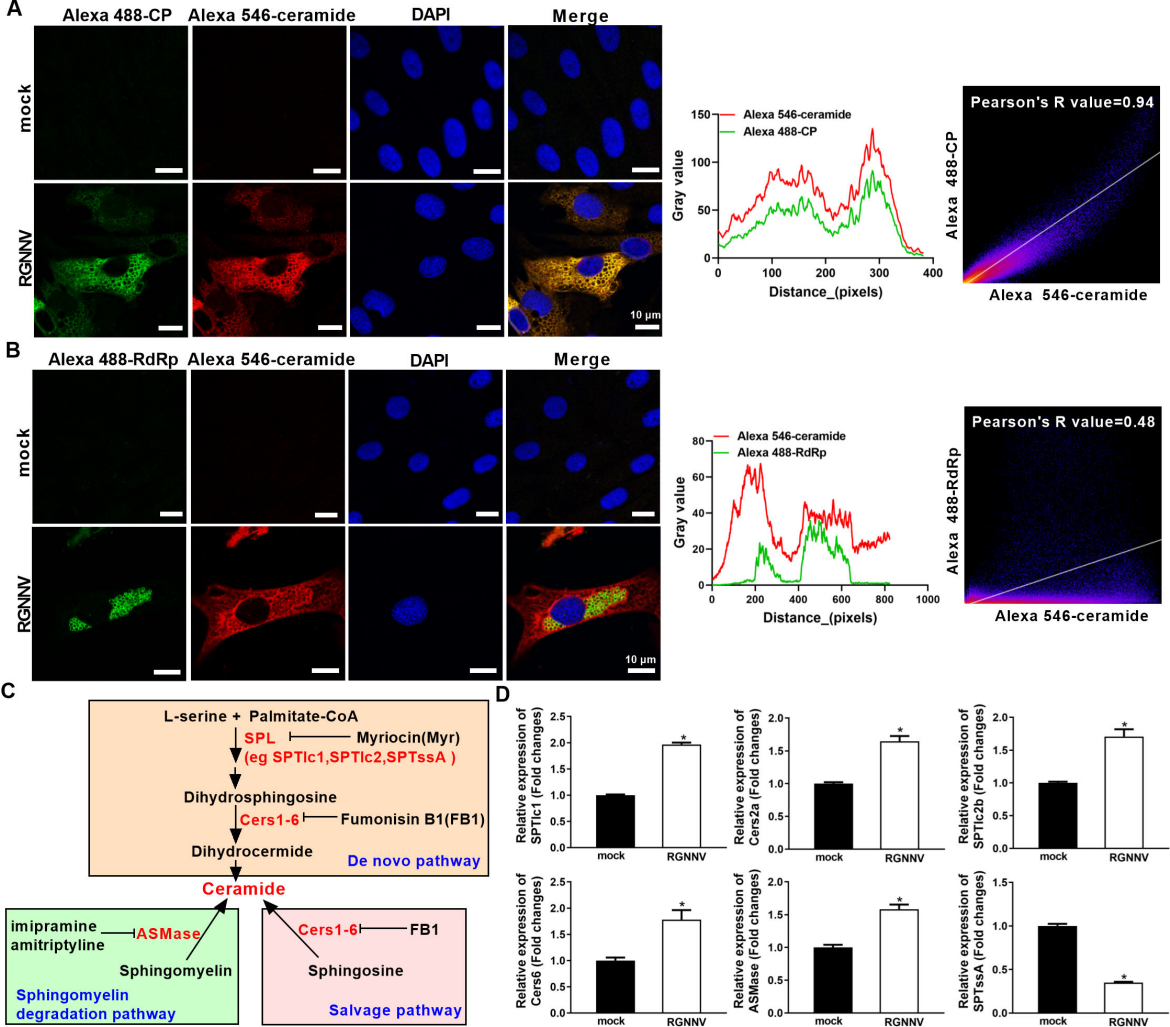
RGNNV infection triggered the ceramides accumulation. (**A, B**) GS cells were infected with RGNNV (MOI = 2) and then were fixed, subjected to IFA, and imaged under an inverted fluorescence microscope. Green signals represented CP (**A**) or RdRp (**B**), red signals represented ceramides, blue staining indicated the nucleus, and yellow signals in the merged image showed that CP was colocalized with ceramide (original magnification, ×100; oil immersion objective). Scale bar = 10 µm. The fluorescence intensity and Pearson’s correlation coefficient for green and red signals were analyzed by Image J. (**C**) Schematic diagram of the key metabolic genes and the inhibitors used in three ceramide synthesis pathways. (**D**) GS cells were infected with RGNNV (MOI = 2) and collected to detect the transcription level of host ceramide synthesis genes, including SPTssA, SPTlc1, SPTlc2b, Cers2a, Cers6, and ASMase by qPCR. *n* = 3, mean ± SD. *P < 0.05.

Given that ceramides were key intermediates for the biosynthesis of other SP species, such as SM and glycosylceramides, or the production of FAs through its hydrolysis (28), we speculated that the enriched ceramides might play crucial roles in RGNNV infection. To test our hypothesis, we firstly assessed the ceramides accumulation in RGNNV-infected cells using a fluorescent anti-ceramide antibody by immunofluorescence assay (IFA). As shown in Fig. 3A and B, weak red fluorescence of ceramides was observed in mock-infected cells, while the obvious enhancement of red fluorescence signals was observed in RGNNV-infected cells. Interestingly, the red signals of ceramides were co-located with the green signals of RGNNV CP, but not with that of RdRp, indicating that the induced ceramides accumulation *in vitro* may be mediated by RGNNV CP. Endogenous ceramide synthesis occurs through the *de novo* biosynthesis, salvage, and sphingomyelin degradation pathways, which are mediated by the key metabolic genes, including serine palmitoyltransferase long chain base subunit 1 (SPTlc1), SPTlc2, SPT small subunit A (SPTssA), ceramide synthase 1–6 (Cers1-6), and acid sphingomyelinase (ASMase) (Fig. 3C) (29); thus, we determined the mRNA expression levels of six genes related to ceramide synthesis in RGNNV-infected cells using quantitative real-time PCR (qPCR). Except for SPTssA, the transcription levels of the other five genes were increased in response to RGNNV infection (Fig. 3D). Thus, we speculated that RGNNV infection triggered the ceramides accumulation *in vitro*.

### Overexpression of RGNNV CP increased the abundance of ceramides

**Fig 4:**
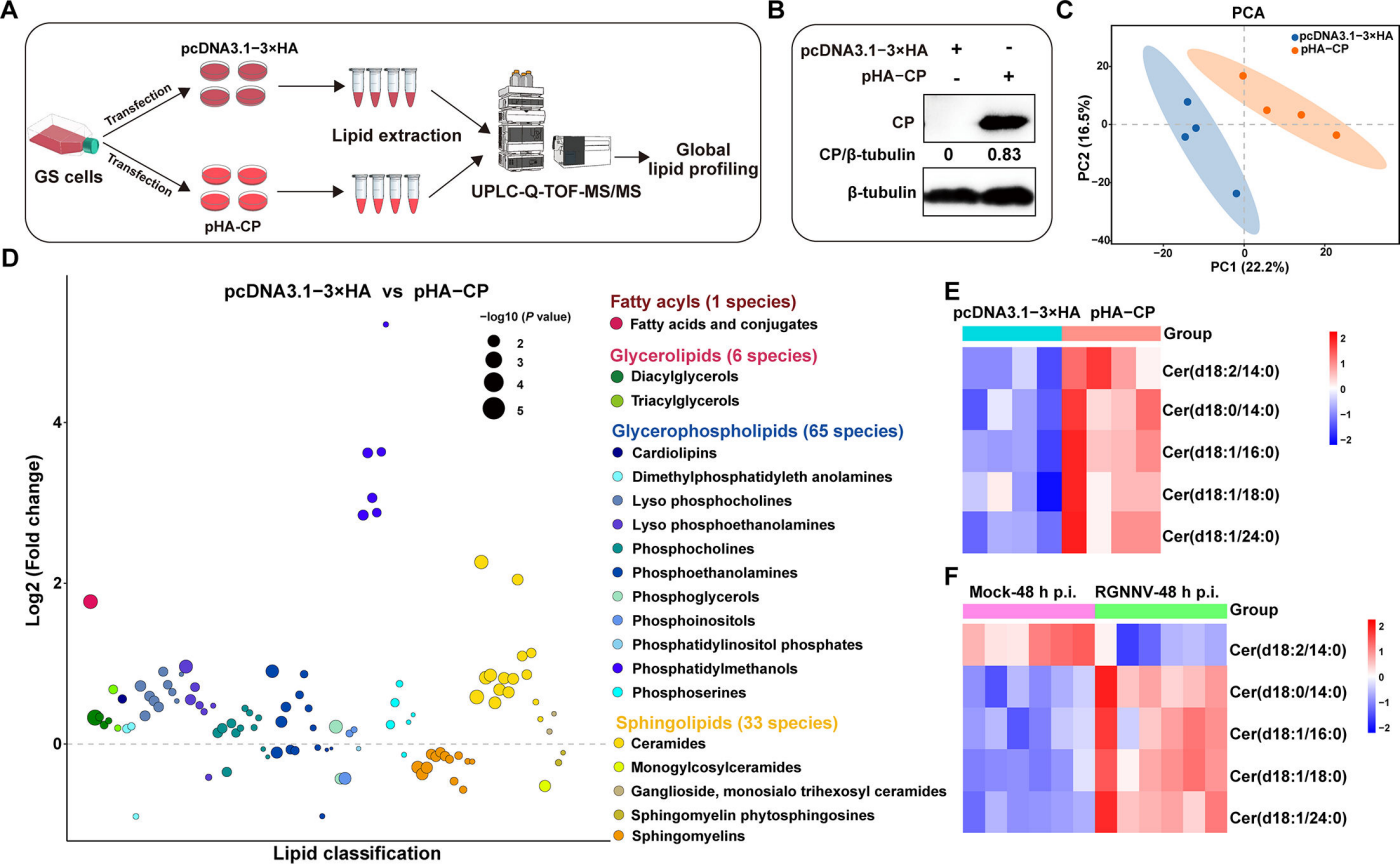
CP overexpression increased the abundance of ceramide species in GS cells. (**A**) Overview and workflow of the global lipidomics in pHA-CP-overexpressing cells. (**B**) The protein expression of CP in GS cells. GS cells (*n* = 4) were transfected with pHA-CP or pcDNA3.1-3×HA for 48 h and were harvested to determine the protein expression of CP by western blotting assay. (**C**) PCA analysis. (**D**) Bubble plots of log2(fold change) in abundance of lipid species in pHA-CP-overexpressing cells relative to pcDNA3.1-3×HA-overexpressing cells at 48 h. Bubble size represents *P* value from *t*-test. (**E, F**) Heatmaps of five ceramide species in CP-overexpressing cells (**E**) and RGNNV-infected grouper cells (**F**). Data were normalized by z-score and subjected to hierarchical clustering. The color gradient from blue (negative z-score, low expression) to red (positive z-score, high expression) indicated the degree of expression deviation.

Given that RGNNV CP was involved in virus-induced autophagy in grouper cells (30), and RGNNV-induced ceramides were co-located with CP, we speculated that CP alone might regulate lipid metabolism *in vitro*. To verify this hypothesis, we analyzed the lipid metabolic profiles of CP-overexpressing cells at 48 h post-transfection (Fig. 4A). As shown in Fig. 4B, CP was successfully expressed in CP-overexpressing cells. PCA results showed that the CP-overexpressing cells clustered together and were separated from control vector cells (Fig. 4C). Compared with control vector cells, 105 differential lipid metabolites were identified in CP-overexpressing cells and categorized into four classes: GPs (61.90%), SPs (31.43%), FAs (0.95%), and GLs (5.71%) (Fig. 4D; Table S2). Of these, the abundances of all Cer, FA, DAG, TAG, ganglioside, monosialo trihexosyl ceramides (GM), lyso PC (LPC), phosphatidylmethanols (PMe), and cardiolipins (CL), as well as almost PS, PC, PE, lyso PE (LPE), and dimethylphosphatidylethanolamines (dMePE), were increased, whereas the majority of SM, sphingomyelin phytosphingosines (phSM), monoglycosylceramides (CerG), and phosphatidylinositol phosphates (PIP) were decreased (Table S2; Fig. S3). Specifically, among the 15 upregulated ceramides in CP-overexpressing cells, five Cer species—Cer(d18:2/14:0), Cer(d18:0/14:0), Cer(d18:1/16:0), Cer(d18:1/18:0), and Cer(d18:1/24:0)—were also increased during RGNNV infection in grouper cells (Fig. 4E and F). Thus, we speculated that CP alone could partially alter lipid metabolism and increase the abundance of host ceramides during RGNNV infection.

### Inhibition of *de novo* ceramide biosynthesis or salvage pathways suppressed RGNNV infection

**Fig 5:**
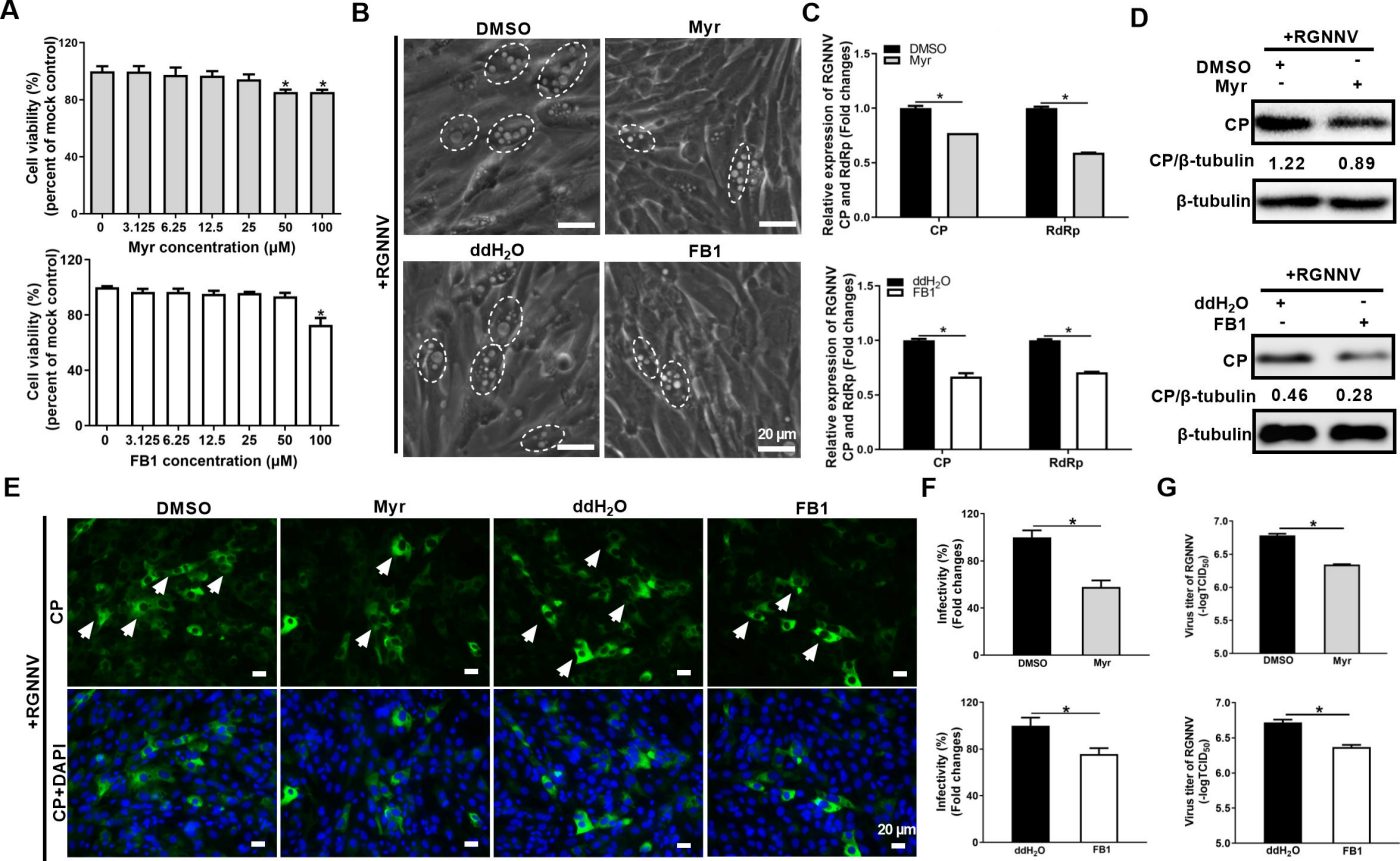
RGNNV infection was reduced upon inhibiting the *de novo* ceramide biosynthesis or salvage pathways. (**A**) Cell viability assay of Myr or FB1 on GS cells. GS cells were pretreated with vehicle control (DMSO or ddH_2_O), or specific inhibitors (Myr or FB1) at 3.125, 6.25, 12.5, 25, 50, or 100 μM, and cell viability was determined using a CCK-8 assay at 48 h. (**B**) The effects of Myr or FB1 on the severity of CPEs induced by RGNNV. The white dashed circles showed that the cells containing vacuoles were induced by RGNNV infection. Scale bar = 20 µm. (**C**) Myr or FB1 reduced the transcription levels of RGNNV CP and RdRp. (**D**) Myr or FB1 reduced the protein level of RGNNV CP by WB assay. (**E, F**) IFA assay of CP protein after treatment with Myr or FB1. The green signals represented RGNNV CP, and the blue staining indicated the nucleus (original magnification, ×40). The arrows showed the green fluorescence signals of CP. Scale bar = 20 µm (**E**). The infectivity was quantified as the percentage of Myr- or FB1-treated cells with incorporated viruses relative to that of control cells which was set as 100% (**F**). (**G**) The effects of Myr or FB1 on the viral titers of RGNNV. *n* = 3, mean ± SD. *P < 0.05.

Our data showed that RGNNV infection triggered ceramides accumulation, while the specific ceramide synthesis pathways involved in RGNNV infection remained unknown. In order to evaluate the roles of ceramide pathways on RGNNV infection, we used the small-molecule inhibitors, including myriocin (Myr; a selective inhibitor of SPT) and fumonisin B1 (FB1; a potent inhibitor of Cers) to inhibit *de novo* ceramide synthesis and salvage pathways (Fig. 3C) (31). After determining the cell cytotoxicity of two inhibitors on GS cells, the non-cytotoxic doses of 25 μM Myr or 50 μM FB1 were used to pretreat cells before RGNNV infection (Fig. 5A; Fig. S4A and B). The effects of two inhibitors on RGNNV infection were examined by qPCR, western blotting (WB), IFA, and virus titers assay. As shown in Fig. 5B, the severity of cytopathic effect (CPE) evoked by RGNNV was weakened in the Myr- or FB1-treated cells compared to that of the DMSO- or ddH_2_O-treated GS cells. Consistently, the addition of Myr or FB1 significantly inhibited the transcription levels of RGNNV RdRp and CP genes (Fig. 5C), as well as the protein level of CP (Fig. 5D). IFA indicated that Myr or FB1 treatment caused reductions in CP-positive cells compared with DMSO or ddH_2_O (Fig. 5E and F). Moreover, the viral titers were decreased in Myr- or FB1-treated cells (Fig. 5G).

**Fig 6:**
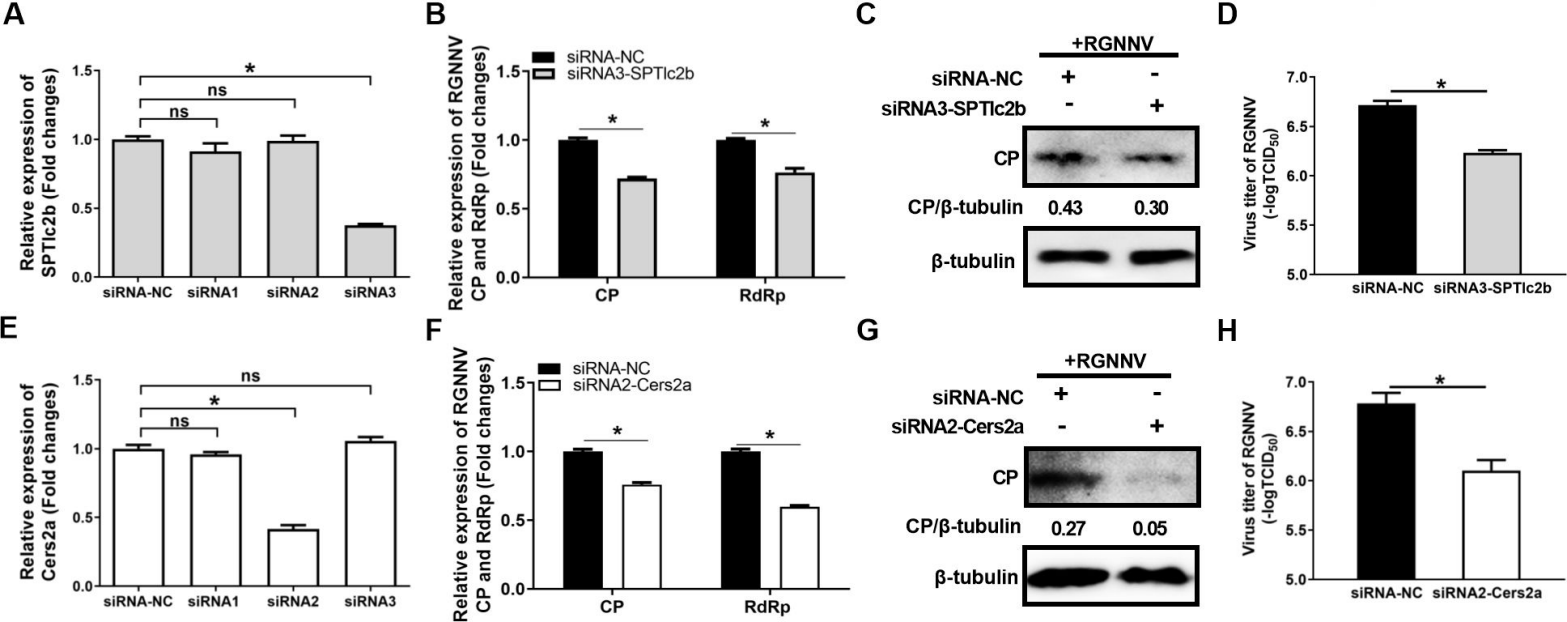
Knockdown of SPTlc2b or Cers2a inhibited RGNNV infection. (**A, E**) The interference efficiency of siRNA-SPTlc2b or siRNA-Cers2a. GS cells were transfected with three specific siRNAs targeting SPTlc2b or Cers2a, or siRNA-NC, and the interference efficiency was determined by qPCR. (**B–H**) siRNA3-SPTlc2b or siRNA2-Cers2a decreased the mRNA levels of RGNNV CP and RdRp (**B, F**), the protein expression of CP (**C, G**), and the viral titers (**D, H**). GS cells transfected with siRNA3-SPTlc2b, siRNA2-Cers2a, or siRNA-NC were infected with RGNNV (MOI = 2) and collected at 24 h p.i. for qPCR (**B, F**), western blotting (**C, G**), and virus titer assay (**D, H**). *n* = 3, mean ± SD. ns, not significant; *P < 0.05.

To further clarify the role of key metabolic enzymes in the d*e novo* ceramide biosynthesis or salvage pathways during RGNNV infection, three specific small interfering RNA (siRNA) oligonucleotides targeting SPTlc2b or Cers2a were designed, and the interference efficiency were evaluated by qPCR. As shown in Fig. 6A and E, compared with Stealth RNAi Negative Control Medium GC Duplexes (siRNA-NC), only siRNA3-SPTlc2b or siRNA2-Cers2a could significantly reduce the expression of SPTlc2b or Cers2a, respectively. Therefore, siRNA3-SPtlc2b and siRNA2-Cers2a were selected for subsequent experiments. qPCR results showed that knockdown of SPTlc2b or Cers2a significantly reduced the transcription levels of RGNNV CP and RdRp genes (Fig. 6B and F). Consistently, compared with siRNA-NC, siRNA3-SPTlc2b or siRNA2-Cers2a significantly inhibited the protein expression of RGNNV CP (Fig. 6C and G) and decreased the viral titers (Fig. 6D and H). Thus, our results showed that inhibition of *de novo* ceramide biosynthesis or salvage pathways suppressed RGNNV infection.

### Inhibition of sphingomyelin-ceramide pathway decreased RGNNV infection

**Fig 7:**
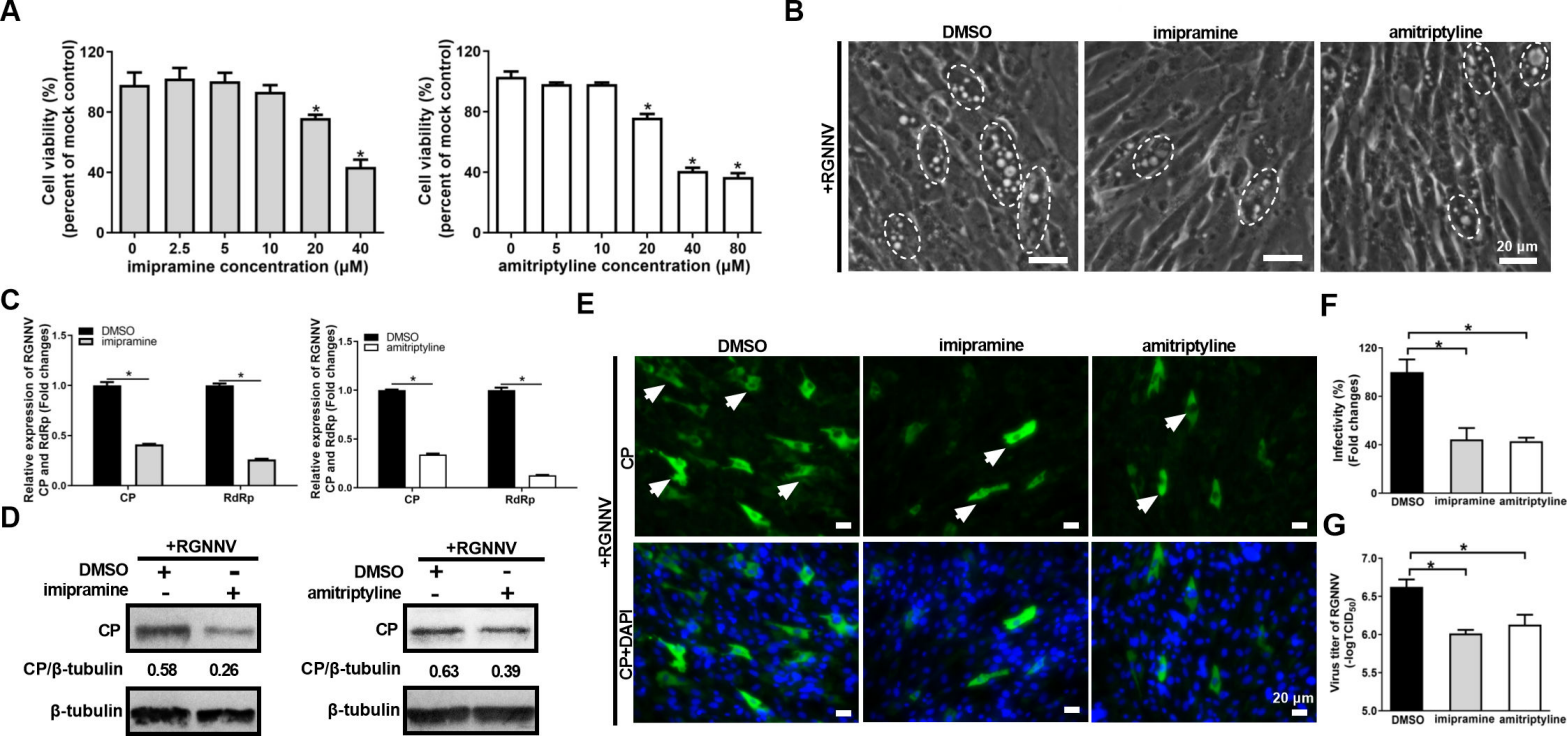
Inhibiting ceramide synthesis via sphingomyelin degradation pathway decreased RGNNV infection. (**A**) Cell viability assay of imipramine or amitriptyline on GS cells. GS cells were pretreated with DMSO, imipramine at 2.5, 5, 10, 15, 20, or 40 μM, or amitriptyline at 5, 10, 20, 40, or 80 μM for 48 h, and cell viability was determined using a CCK-8 assay. (**B**) Imipramine or amitriptyline weakened the severity of CPEs. The white dashed circles showed that the cells containing vacuoles were induced by RGNNV infection. Scale bar = 20 µm. (**C**) Imipramine or amitriptyline reduced the transcription expression of RGNNV CP and RdRp. (**D**) Imipramine or amitriptyline reduced the protein level of RGNNV CP by WB assay. (**E, F**) The CP protein expression by IFA assay after treatment with imipramine or amitriptyline. Green signals represented RGNNV CP, and blue staining indicated the nucleus (original magnification, ×40). The arrows showed the green fluorescence signal of CP. Scale bar = 20 µm (**E**). The infectivity was quantified as the percentage of imipramine- or amitriptyline-treated cells with incorporated viruses relative to that in DMSO-treated cells which was set as 100% (**F**). (**G**) Imipramine or amitriptyline reduced the viral titers of RGNNV. *n* = 3, mean ± SD. *P < 0.05.

Except for *de novo* biosynthesis and salvage pathways, ceramide synthesis occurs through sphingomyelin degradation pathway that ceramides can be synthesized from sphingomyelin by SMase (29). Our lipidomic data revealed that the abundance of all SMs was enriched in RGNNV-infected cells at a later stage of virus infection; thus, we hypothesized that ceramides accumulation induced by RGNNV infection was driven through the sphingomyelin degradation pathway. To address this question, GS cells were pretreated with two ASMase inhibitors, including imipramine and amitriptyline, before RGNNV infection, and the effects of two inhibitors on viral infection were investigated. As shown in Fig. 7A and Fig. S4C, a counting kit-8 (CCK-8) assay confirmed that 10 μM imipramine and 10 μM amitriptyline were not toxic in GS cells. In imipramine- or amitriptyline-treated cells, the severity of CPE induced by RGNNV infection was both reduced compared with DMSO-treated cells (Fig. 7B). Furthermore, the mRNA levels of RdRp and CP were significantly inhibited by imipramine or amitriptyline (Fig. 7C). In addition, both imipramine and amitriptyline significantly decreased the protein expression of CP (Fig. 7D through F). Furthermore, compared with the DMSO-treated cells, the viral titers were both decreased in imipramine- or amitriptyline-treated cells (Fig. 7G). Taken together, three ceramide synthesis pathways were all essential for RGNNV infection.

### Exogenous addition of C16-ceramide promoted RGNNV infection

**Fig 8:**
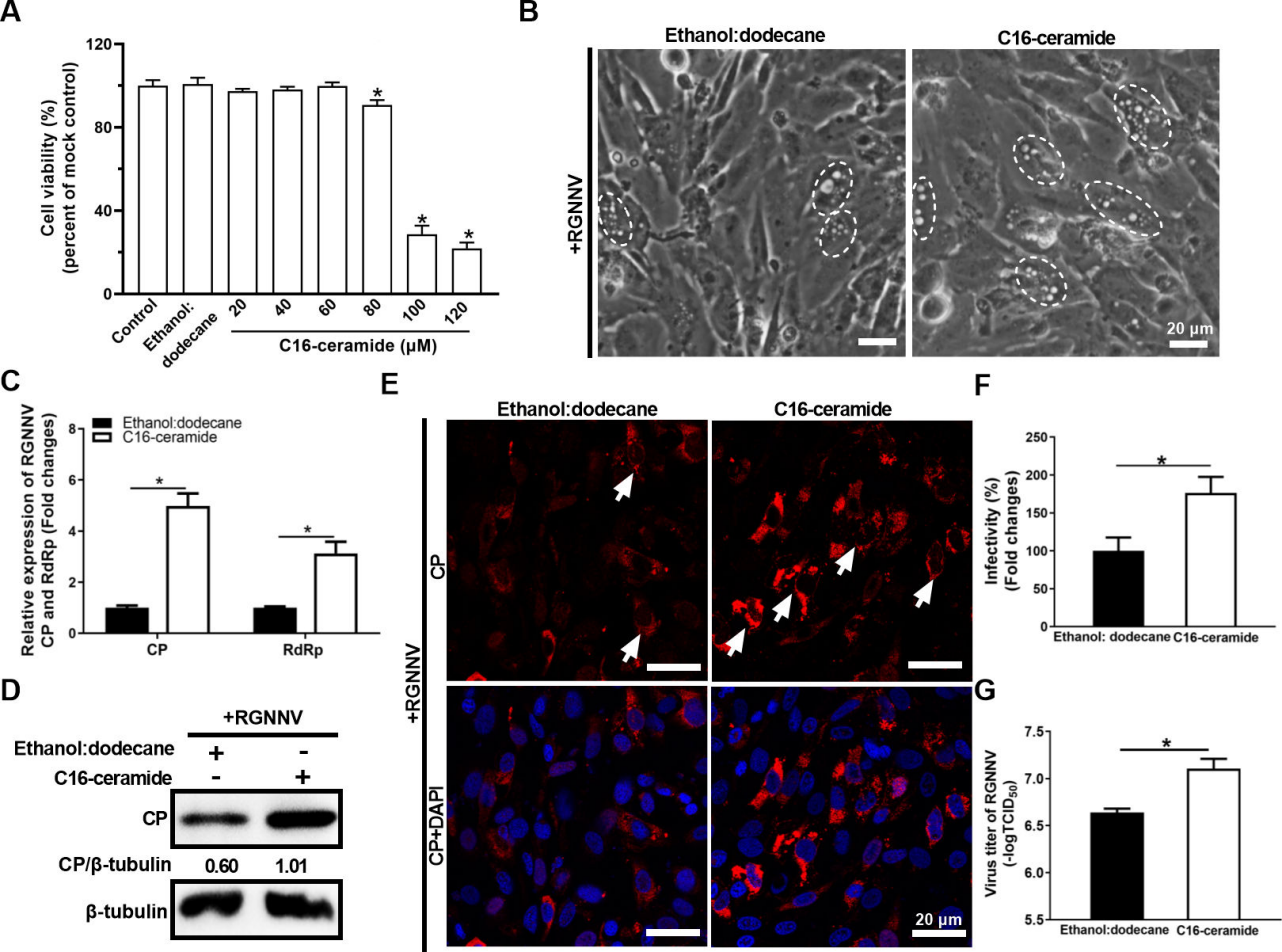
C16-ceramide (d18:1/16:0) promoted RGNNV infection. (**A**) Cell viability assay of C16-ceramide (d18:1/16:0) on GS cells. GS cells were pretreated with ethanol:dodecane (98:2, vol/vol; 0.05% final concentration) or C16-ceramide (d18:1/16:0) at 20, 40, 60, 80, 100, or 120 μM for 48 h, and cell viability was determined using a CCK-8 assay. (**B–G**) C16-ceramide (d18:1/16:0) promoted RGNNV infection. GS cells were pretreated with 60 μM C16-ceramide (d18:1/16:0) or ethanol:dodecane (98:2, vol/vol) and infected with RGNNV (MOI = 2) for 24 h. The severity of CPEs (**B**) induced by RGNNV infection was observed at a phase contrast microscope. The white dashed circles showed that the cells containing vacuoles were induced by RGNNV infection. Scale bar = 20 µm. Besides, the RGNNV-infected cells were harvested for qPCR (**C**), WB (**D**), IFA (**E, F**), and virus titer (**G**) assay. Red signals represented RGNNV CP, and blue staining indicated the nucleus (original magnification, ×40). The arrows showed the red fluorescence signals of CP. Scale bar = 20 µm (**E**). The infectivity was quantified as the percentage of C16-ceramide-treated cells with incorporated viruses relative to that of ethanol:dodecane (98:2, vol/vol)-treated cells which was set as 100% (**F**). (**G**) The virus titers of RGNNV were increased in C16-ceramide-treated cells. *n* = 3, mean ± SD. *P < 0.05.

Given that ceramide (d18:1/16:0) was one of the identified ceramides in RGNNV-infected cells and CP-overexpressing cells (Fig. 4E and F), we further explored the effects of C16-ceramide on RGNNV infection. GS cells were pretreated with C16-ceramide (d18:1/16:0), a synthetic ceramide analog, and then infected with RGNNV. At 24 h p.i., the cells were collected to detect RGNNV infection by qPCR, WB, IFA, and virus titers assay. As shown in Fig. 8A and Fig. S4D, a CCK-8 assay, confirmed that there was no significant reduction in cells treated with less than 60 μM C16-ceramide (d18:1/16:0) compared with control or ethanol:dodecane-treated cells. The severity of CPE induced by RGNNV was enhanced by C16-ceramide (d18:1/16:0) (Fig. 8B). Next, qPCR assay showed that compared with ethanol:dodecane-treated cells, the transcription levels of RGNNV CP and RdRp genes were increased in C16-ceramide (d18:1/16:0)-treated cells (Fig. 8C). Consistently, compared with control, treatment with C16-ceramide significantly upregulated the protein expression and the positive red signals of CP (Fig. 8D through F). These results were consistent with the increased viral titers in C16-ceramide-treated cells (Fig. 8G). Thus, the promoting role of ceramide in RGNNV infection was confirmed.

### The inhibitory effects of Myr or imipramine on RGNNV infection were rescued by exogenous addition of C16-ceramide

**Fig 9:**
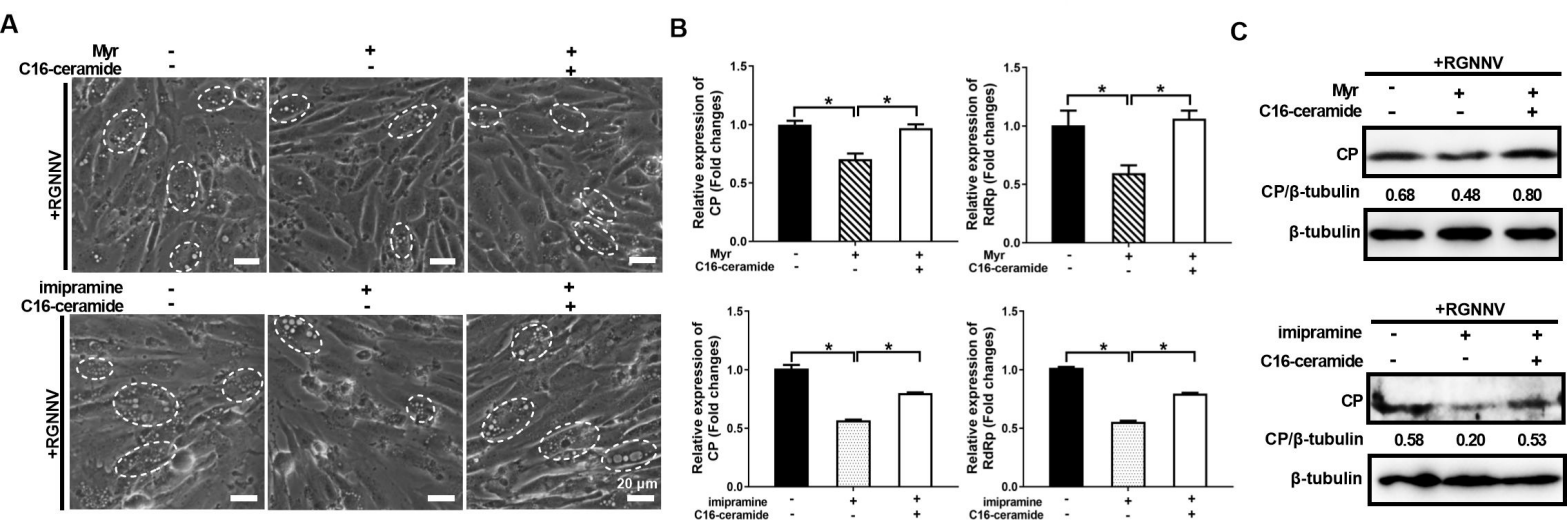
The inhibitory effects of Myr or imipramine on RGNNV infection were saved by exogenous addition of C16-ceramide (d18:1/16:0). (**A–C**) GS cells were pretreated with Myr (25 μM) or imipramine (10 μM) for 2 h and then treated with C16-ceramide (d18:1/16:0) (60 μM) for 24 h. After being infected with RGNNV for 24 h, cells were collected to determine the severity of CPEs (**A**), viral genes transcription (**B**), and protein (**C**) levels. (**A**) The effects of Myr or imipramine alone or with C16-ceramide on the severity of CPEs induced by RGNNV at 24 h p.i. The white dashed circles showed that the cells containing vacuoles were induced by RGNNV infection. Scale bar = 20 µm. (**B**) The reduced transcription expression of RGNNV CP and RdRp at 24 h p.i. by Myr or imipramine was saved by exogenous addition of C16-ceramide. (**C**) The effects of Myr or imipramine alone or with C16-ceramide on the protein expression of RGNNV CP. *n* = 3, mean ± SD. *P < 0.05.

Given that inhibiting ceramide synthesis pathways blocked RGNNV infection, we further investigated whether the inhibitory effects of two inhibitors, including Myr or imipramine, could be saved by the exogenous addition of C16-ceramide (d18:1/16:0). As shown in Fig. 9, compared with DMSO, treatment with either Myr or imipramine weakened the severity of vacuoles induced by RGNNV infection and decreased viral gene transcription and protein expression. Notably, compared with the control cells, the weakened severity of CPE induced by RGNNV in Myr- or imipramine-treated cells was partly reversed in C16-ceramide-treated cells (Fig. 9A). Besides, the reduced mRNA levels of RGNNV CP and RdRp were also reversed by exogenous addition of C16-ceramide (Fig. 9B). Consistently, exogenous addition of C16-ceramide rescued the decreased protein expression of CP in Myr- or imipramine-treated cells (Fig. 9C). Thus, exogenous addition of C16-ceramide could rescue the inhibitory effects of inhibiting ceramide synthesis pathways on RGNNV infection.

### Exogenous addition of C16-ceramide increased RGNNV-induced autophagy

**Fig 10:**
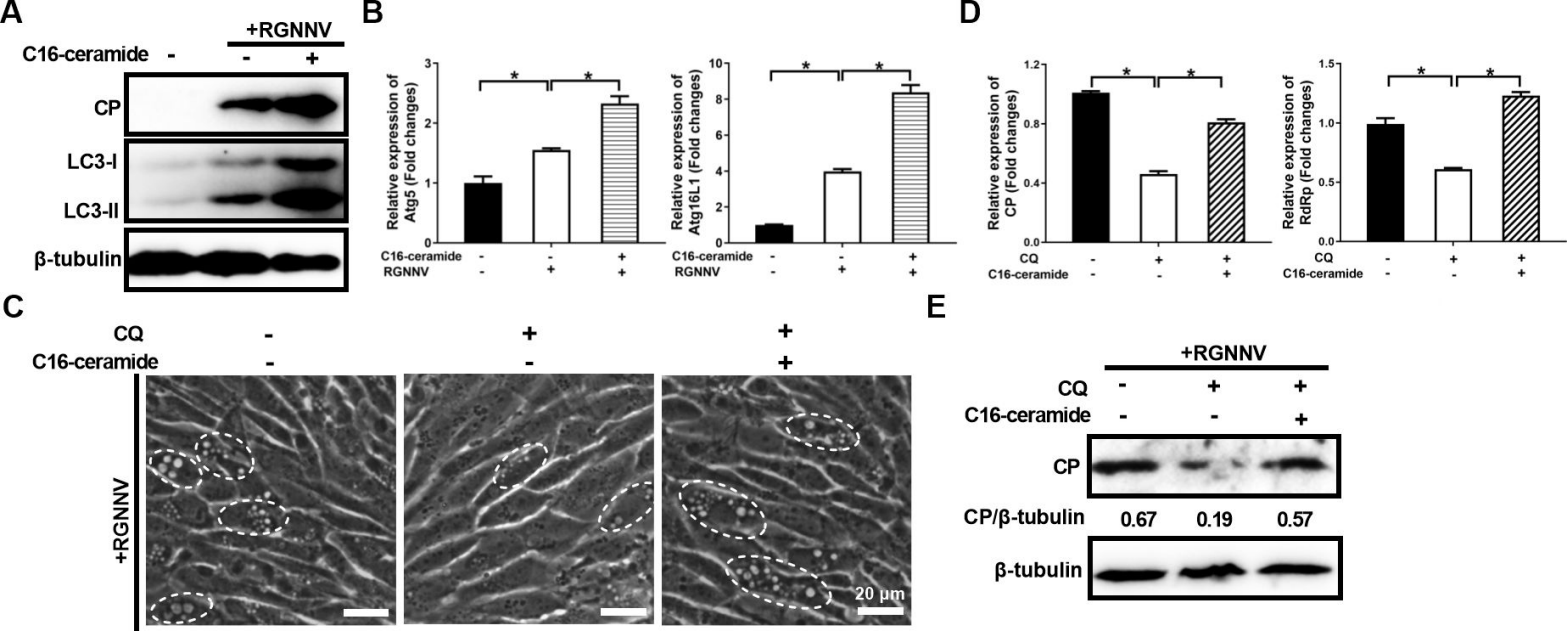
C16-ceramide (d18:1/16:0) promoted RGNNV infection by inducing autophagy. (**A, B**) C16-ceramide (d18:1/16:0) promoted the protein expression of LC3, and the transcription levels of Atg genes induced by RGNNV. GS cells were infected or not with RGNNV (MOI = 2), in the presence or absence of C16-ceramide (60 μM), and were collected for WB (**A**) or qPCR (**B**) assay. (**C–E**) C16-ceramide (d18:1/16:0) inhibited the antiviral effects of CQ on RGNNV infection. GS cells were treated with CQ (5 μM) in the presence or absence of C16-ceramide (60 μM) and then infected with RGNNV (MOI = 2). The severity of CPEs induced by RGNNV infection was observed at a phase contrast microscope. The white dashed circles showed that the cells containing vacuoles were induced by RGNNV infection. Scale bar = 20 µm (C). Besides, the RGNNV-infected cells were harvested for qPCR (**D**) or WB (**E**) assay. *n* = 3, mean ± SD. *P < 0.05.

As a central molecule in sphingolipid metabolism, ceramides play important roles in regulating cell death (32). A previous study revealed that RGNNV induced autophagy to promote viral infection (14). To investigate the potential mechanisms by which ceramides promote viral infection, GS cells pretreated with C16-ceramide (d18:1/16:0) were infected with RGNNV. Mock- or infected cells were then harvested to detect the protein expression of LC3 and the mRNA expression of autophagy-related genes (Atg). As shown in Fig. 10A, compared with mock-infected cells, the protein expression of LC3-II was increased in the RGNNV-infected cells, and these were enhanced by C16-ceramide (d18:1/16:0) treatment. Similarly, the transcription of Atg genes, including Atg5 and Atg16L1, was significantly induced by RGNNV, and exogenous addition of C16-ceramide (d18:1/16:0) further enhanced the promotion (Fig. 10B).

To further explore the impact of C16-ceramide on autophagy, we determined autophagy flux status using chloroquine (CQ), an inhibitor of autophagosome-lysosome fusion. As shown in Fig. 10C through E, compared with control cells, treatment with CQ inhibited vacuole fusion during RGNNV infection and decreased viral gene transcription and protein expression, in agreement with the previous study (12). However, this alleviating vacuole fusion was reduced in the presence of C16-ceramide (Fig. 10C). Consistently, the decreased levels of viral genes mRNA and CP protein were also rescued by C16-ceramide (Fig. 10D and E). Taken together, C16-ceramide promoted RGNNV infection via increasing RGNNV-induced autophagy.

## DISCUSSION

It is well-known that viruses can regulate and utilize host lipid metabolism at various stages in the viral life cycle to facilitate their replication (33). As a central hub of sphingolipid metabolism, ceramide exerts different actions on virus replication (20). Most (+) RNA viruses, including flaviviruses, nodaviruses, and bromoviruses, induced the rearrangement of organelle membrane, resulting in the alteration of host lipid metabolism (5). RGNNV, belonging to the genus *Betanodavirus*, also induced the alterations of the membrane structures and the formation of cytoplasmic vacuolization *in vitro* (12, 17). However, whether the membrane remodeling induced by RGNNV altered the total lipid profiles and the roles of the critical lipid components on virus replication still remained uncertain.

Here, we investigated the global lipidomic profiles of RGNNV infection in grouper cells. Our results showed that RGNNV infection significantly altered the abundance of lipid metabolites, spanning GPs, FAs, GLs, STs, and SPs. Notably, the majority of the metabolites belonging to the subclass of glycerophospholipids, including PC and PI, were markedly upregulated upon RGNNV infection. As the primary components of most cellular membranes, PC was also increased during DENV (6, 7). In addition, GPs were required for active complete replication of FHV, another member in the family *Nodaviridae* (8), and membrane rearrangement in replication complex assembly was essential for viral replication *in vitro* (4). Given that RGNNV infection induced the formation of cytoplasmic vacuolization (12), we speculated that the remodeling of intracellular membrane induced by RGNNV might be mainly due to the alteration of GPs during viral infection.

Except for the altered GPs, the abundances of most TAG in RGNNV-infected cells were higher than that in mock-infected cells. Given the storage of TAG in lipid droplets (27) and the induction of lipid droplet formation by RGNNV infection (17), it was speculated that RGNNV induced TAG to form lipid droplets for infection. Our previous metabolic analysis showed that representative metabolites in *de novo* lipogenesis, such as palmitic acid and oleic acid, were significantly increased in RGNNV-infected cells, and further study confirmed the essential roles of cellular fatty acid synthesis in virus infection (17). Here, lipidomic analysis further indicated that most of the eicosanoids, docosanoids, octadecanoids, and fatty esters levels were elevated in RGNNV-infected cells. These lipid metabolites could exert anti-inflammatory, antioxidant, and other immune modulatory effects and usually were manipulated upon virus infection (34, 35). For instance, DENV infection increased the levels of three prostaglandins, one thromboxane, and two docosanoid substances (resolvin d5 and epoxy-DHA) (7). Whether these lipid metabolites exerted immune modulatory effects on RGNNV infection needed further investigation.

Sphingolipids, known as the structural components of mammalian cell membranes, can regulate various cellular signaling pathways, such as cell growth, differentiation, death, apoptosis, autophagy, and immune response (36). During DENV infection, the abundances of several SPs, such as sphinganine, sphinganine-1-PC, sphingosine, Cer, and hexosylceramide, were increased, but that of SM remained unchanged (7). In our study, the abundances of all the detected SM and NGSL, as well as most of ceramides and AGSL, were elevated at the late stage of RGNNV infection. Notably, ectopic expression of RGNNV CP alone also partially altered the abundances of SPs, especially increasing the levels of ceramides. Among them, five Cer species—Cer(d18:2/14:0), Cer(d18:0/14:0), Cer(d18:1/16:0), Cer(d18:1/18:0), and Cer(d18:1/24:0)—were consistently increased with their changes during RGNNV infection, demonstrating the potential role of CP on modulating ceramide metabolism. Similarly, both ZIKV infection and ectopic expression of ZIKV NS4B protein significantly enriched ceramides, and sphingolipid ceramides were even redistributed to virus replication sites, suggesting that ceramide flux took part in the formation of virus replication complexes (23). Notably, here, we also found that ceramides were co-localized with viral structural protein CP during RGNNV infection. In addition, the relative expression levels of key genes involved in ceramide synthesis were also upregulated during RGNNV infection. Thus, we speculated that ceramide exerted crucial roles during RGNNV infection.

As important bioactive lipids, ceramides are produced through three distinct pathways—the *de novo*, sphingomyelin degradation, and salvage pathways—and function as a hub in sphingolipid metabolism (29). Interestingly, ceramides have been demonstrated to participate in different stages of virus infection but showed the opposite effects (enhancement or inhibition) on the replication of different viruses (20). The accumulation of ceramides in IAV-infected cells was mainly driven through the *de novo* biosynthesis, and inhibiting the *de novo* ceramide pathway enhanced IAV replication (24). Differently, Myr or FB1 treatment inhibited the replication of WNV and ZIKV but enhanced that of DENV or rotaviruses (RVs) (22, 23, 26). Depletion of ceramides through the sphingomyelin degradation pathway reduced the replication and release of ZIKV and severe acute respiratory syndrome-related coronavirus 2 (SARS-CoV-2), but not IAV (37, 38). Addition of exogenous C2- or C18-ceramide promoted human norovirus (HuNoV) replication, whereas C6-ceramide, C16-ceramide, C18-ceramide, or C24-ceramide inhibited IAV or RV infection (24, 26, 39), indicating that the regulatory effects of different ceramides on various viral infection were specific. In the current study, RGNNV infection was significantly decreased upon pharmacological inhibitors or knockdown of enzymes involved in *de novo* biosynthesis, salvage, or sphingomyelin degradation pathways of ceramide synthesis. Moreover, the inhibitory effects were significantly rescued by the addition of C16-ceramide, suggesting that ceramides were essential for RGNNV infection *in vitro*.

Mechanistically, ceramides have been found to be responsible for diverse cellular functions, including apoptosis, autophagy, inflammation, fatty acid oxidation, and endoplasmic reticulum stress response (40, 41). Moreover, virus-induced cell death could also be mediated by ceramides, thereby affecting virus infection (26). For example, adenovirus (ADV) infection triggered the accumulation of ceramides, which in turn induced autophagy and apoptosis (42). Blocking ceramide synthesis in the *de novo* and salvage pathways increased cell death in ZIKV and the apathogenic vaccine strain of yellow fever virus (YFV) infection and then significantly enhanced virus production (43). Besides, during Epstein-Barr virus (EBV) infection, C6-ceramide induced accumulation of the autophagosome marker LC3-II, and CQ, an inhibitor of autophagosome-lysosome fusion, increased LC3-II accumulation both before and after C6-ceramide treatment (21). Our previous studies found that RGNNV infection induced autophagy (14), and CQ inhibited RGNNV infection (12). Here, our data indicated that the addition of C16-ceramide enhanced RGNNV-induced autophagy, including the mRNA expressions of autophagy-related genes and the protein level of LC3-II. Moreover, treatment with C16-ceramide restored RGNNV infection under the treatment of CQ. Together, we speculated that ceramides promoted RGNNV infection by triggering autophagy.

In conclusion, we described the first global lipidomic profiles of fish nodavirus infection *in vitro*. Our data demonstrated that RGNNV infection significantly altered the lipid metabolism; in particular, RGNNV manipulated the ceramide metabolism via CP protein. Consistent with the accumulation of ceramides in RGNNV-infected cells, three major ceramide synthesis pathways, including *de novo* biosynthesis, salvage, and sphingomyelin degradation, were all required for RGNNV infection. Moreover, the exogenous C16-ceramide significantly promoted RGNNV infection via the regulation of RGNNV-induced autophagy. Together, our findings not only contributed greatly to understanding the crucial roles of bioactive lipids in fish nodavirus infection, but also shed new light on ceramides as part of therapeutic strategies for controlling and treating fish viral diseases.

## MATERIALS AND METHODS

### Cells and virus

GS cells used in the study were cultured in Leibovitz’s L15 medium supplemented with 10% fetal bovine serum (FBS, Gibco) at 28°C (44). The RGNNV was prepared as described previously (45).

### Non-targeted lipidomic analysis of RGNNV-infected and CP-overexpressing cells *in vitro*

#### Lipid sample collection

On the one hand, GS cells were equally seeded at a 25 cm^2^ cell culture flasks (1 × 10^7^ cells/flask, *n* = 6) for 18 h and infected with RGNNV at a multiplicity of infection (MOI) of 2 or treated with an equivalent volume of medium. After 1 h, cells were replaced with fresh medium and maintained for the indicated time post-infection. At 24 and 48 h p.i., mock- and RGNNV-infected cells were harvested for lipid sample collection as follows. On the other hand, GS cells were seeded into 6-well plates (1 × 10^6^ cells/flask, *n* = 4) and transfected with 6 μg pHA-CP or pcDNA3.1-3×HA per well using Lipofectamine 2000 transfection reagent (Invitrogen). After 48 h post-transfection, GS cells were harvested and centrifuged at 4°C, 300 × *g* for 10 min. After washing once with cold PBS, the cells were centrifuged at 4°C, 300 × *g* for 10 min. Then, the cell pellets were treated with 600 μL methanol:water (1:1, vol/vol) containing 20 μL internal standard (Lyso PC 17:0) and added with 600 μL chloroform for an ultrasound in ice water bath for 10 min. After aspiration of the chloroform layer and vacuum drying, the samples were added with 600 μL chloroform:methanol (2:1, vol/vol), vortexed for 30 s, and lysed by ultrasonic waves for 10 min. The lipid residue was resuspended with 300 μL isopropanol:methanol (1:1, vol/vol), vortexed for 30 s, and followed by an ultrasound for 3 min. After centrifugation at 4°C, 12,000 × *g* for 15 min, 200 μL supernatant was transferred to vials for LC-MS analysis (Shanghai Luming Biotechnology Co., Ltd.). Quality control (QC) samples were obtained from each sample.

#### UPLC-Q-TOF-MS/MS profiling analysis

A Nexera UPLC (Shimadzu, Kyoto, Japan) system interfaced with Q Exactive mass spectrometer (Thermo Fisher Scientific, Waltham, MA, USA) was used for LC-MS/MS analysis. The UPLC conditions were as follows: column temperature, 45°C; mobile phase A, acetonitrile:water (60:40, vol/vol) plus 10 mM ammonium formate and 0.1% formic acid; mobile phase B, acetonitrile:isopropanol (10:90, vol/vol) plus 10 mM ammonium formate and 0.1% formic acid; flow rate, 0.35 mL/min; and injection volume, 5 μL. Gradient conditions were as follows: 0 min, 30% phase B; 3 min, 30% phase B; 5 min, 62% phase B; 15 min, 82% phase B; 16.5 min, 99% phase B; 18 min, 99% phase B; 18.1 min, 30% phase B; and 22 min, 30% phase B. The samples were then analyzed under the positive and negative ionization modes. The mass parameters of positive ionization modes (ESI+) were as follows: heater temperature, 300°C; sheath gas flow rate, 45 arb; aux gas flow rate, 15 arb; sweep gas flow rate, 1 arb; spray voltage, 3.5 KV; capillary temperature, 320°C; S-Lens RF level, 50%; and MS1 scan range, 120–1,800. The mass parameters of negative ionization modes (ESI−) were as follows: heater temperature, 300°C; sheath gas flow rate, 45 arb; aux gas flow rate, 15 arb; sweep gas flow rate, 1 arb; spray voltage, 3.1 KV; capillary temperature, 320°C; S-Lens RF level, 50%; and MS1 scan range, 120–1,800.

#### Data preprocessing and statistical analysis

Data from ESI+ and ESI− were preprocessed using Progenesis QI v2.3 (Nonlinear Dynamics, Newcastle, UK), and the lipids were identified by searching mass per charge ratio (m/z) using the Human Metabolome Database (HMDB), LIPID Metabolites, METLIN, and the local LuMet-Animal3.0 database (46). PCA analysis was performed. The value of each sample represented the total peak area that was normalized to all peak area. Student’s *t*-test was used to calculate the statistically significant threshold of *P* value. Differential metabolites were screened based on the combination of *P* value (< 0.05) and fold change (FC) (>2 or < 1) (47).

### Ceramide and inhibitors

C16-ceramide (d18:1/16:0) was purchased from Avanti Polar Lipid and was dissolved in ethanol:dodecane (98:2, vol/vol; 0.05% final concentration) (48). Myriocin and fumonisin B1 were obtained from MCE and dissolved in sterile DMSO and ddH_2_O, respectively. Imipramine (hydrochloride) and amitriptyline HCl were purchased from APExBIO and dissolved in DMSO according to the manufacturer’s instructions. All stocks were stored at −20°C and diluted in Leibovitz’s L15 medium supplemented with 10% FBS prior to use.

### Cell viability

Cell viability was assessed by CCK-8 (Yuheng, China) following the manufacturer’s protocol. Briefly, GS cells were seeded in 96-well plates for 18 h and then incubated with different experimental concentrations of reagents, DMSO, or ddH_2_O for 48 h. The cells were then washed with fresh normal medium three times and were added with 100 μL Leibovitz’s L15 medium supplemented with 10% FBS. After adding 10 μL CCK-8 into each well, cells were incubated at 28°C for 4 h. Then the absorbance was measured in Varioskan LUX multimode microplate reader (ThermoFisher, USA) at 450 nm.

### Virus infection

For the drug assay, GS cells were seeded in 24-well plates for 18 h and pretreated with C16-ceramide (d18:1/16:0) (60 μM) for 24 h, or with inhibitors of different pathways in ceramide synthesis, including Myr (25 μM), FB1 (50 μM), imipramine (10 μM), and amitriptyline (10 μM), for 2 h at 28°C. The cells were then infected with RGNNV (MOI = 2.0) in the presence of ceramide synthesis inhibitors, and the severity of CPE (the increased or decreased numbers of cells containing vacuoles) induced by RGNNV was photographed using a phase contrast microscope. Besides, cells were collected at 24 h p.i. to determine the expression of viral genes by qPCR, western blotting, IFA, or virus titers assay.

For the knockdown assay, three specific siRNA oligonucleotides targeting different sequences of SPTlc2b or Cers2a were designed and synthesized from GenePharma. The specific siRNA sequences were listed in Table S3. After screening the knockdown efficiency of siRNAs, GS cells were transfected with siRNA-NC and siRNA of Cers2a or SPTlc2b using Lipofectamine 2000 and infected with RGNNV (MOI = 2.0). After 24 h p.i., GS cells were collected for qPCR, western blotting, or virus titers assay.

### RNA isolation and qPCR analysis

Total RNA of mock- and RGNNV-infected cells was extracted using the SV Total RNA Isolation Kit (Promega, USA) and reverse-transcribed using ReverTra Ace qPCR RT Kit (Toyobo, Japan) according to the manufacturer’s instruction. The transcription levels of host ceramide synthesis genes, Atg genes, and viral genes were detected using SYBR Green I Reaction Mix (Toyobo, Japan) in Applied Biosystems QuantStudio 5 Real-Time Detection System (Thermofisher, USA), as described previously (49). The used primers were listed in Table S3. The relative fold changes of targeted genes were normalized to β-actin and calculated using the 2^−ΔΔCT^ method.

### Western blotting assay

Cells were collected at 24 h p.i. and were resuspended in Pierce IP lysis buffer (25 mM Tris-HCl, pH 7.4, 150 mM NaCl, 1 mM EDTA, 1% NP-40, and 5% glycerol). The extracted proteins were separated by 10% SDS-PAGE and transferred to 0.22 μm polyvinylidene difluoride (PVDF) membranes (Millipore, USA). After blocking with 5% (wt/vol) skim milk in PBS containing 0.5% Tween 20 (PBST), the PVDF membranes were incubated with the primary antibodies, including anti-RGNNV CP (1:2,000; prepared in our lab), LC3 (1:1,000; CST, USA), and anti-β-tubulin (1:3,000; Abcam, USA), at room temperature for 2 h. After washing with PBST three times, the membranes were incubated using the horseradish peroxidase (HRP)-conjugated sheep anti-rabbit IgG (1:3,000; Abcam, USA) at room temperature for 2 h. The membranes were visualized using Pierce ECL Western Blotting Substrate (Thermofisher, USA), and the intensity of the CP band was normalized to the expression of β-tubulin.

### Indirect immunofluorescence assay

The protein expressions of CP, RdRp, and ceramide in mock- and RGNNV-infected cells were also evaluated by IFA as previously described (50). The cells were fixed in 4% paraformaldehyde for 1 h, permeabilized with 0.2% Triton X-100 for 15 min, and blocked with 0.2% bovine serum albumin (BSA) for 30 min at room temperature. After washing three times with PBS, GS cells were incubated with rabbit anti-RGNNV CP (1:200; prepared in our lab), rabbit anti-RGNNV RdRp (1:200; prepared in our lab), or mouse anti-ceramide (1:100; Sigma, USA) diluted in 0.2% BSA for 2 h, followed by incubation with Alexa Fluor 546-conjugated anti-rabbit IgG Fab2 (1:200; Invitrogen, USA) or Alexa Fluor 488-conjugated anti-mouse IgG Fab2 (1:200; Cell signaling, USA) for 2 h at room temperature. Finally, the cells were stained with 4,6-diamidino-2-phenylindole (DAPI) and observed under an inverted fluorescence microscope.

### Virus titer assay

To explore the effects of ceramide on the viral titers of RGNNV, the infected cells were harvested at 24 h p.i. for virus titers assay as described previously (51). Briefly, after being freeze–thawed five times, 100 μL of diluted samples from 10^−1^ to 10^−9^ were added to GS cells in a 96-well plate. After 96-144 h p.i., the CPE induced by RGNNV was observed under a light microscope and measured with the 50% tissue culture infectious dose (TCID_50_) assay. Each sample was measured in triplicate.

### Data analysis and statistics

Statistics were performed using one-way ANOVA with SPSS version 20, and differences were considered statistically significant when *P* was < 0.05 (*). Each experiment was repeated three times independently at different time points, and the results were represented as mean ± standard deviation (SD) from one representative experiment.

## Data Availability

The lipid metabolome data have been deposited in the National Genomics Data Center under the accession number PRJCA058502 and are publicly accessible at https://ngdc.cncb.ac.cn/bioproject/browse/PRJCA058502.

## SUPPLEMENTAL MATERIAL

Supplemental material jvi.01991-25-s0001.docx Fig. S1 to Fig. S4; Tables S1 to S3.

ASM does not own the copyrights to Supplemental Material that may be linked to, or accessed through, an article. The authors have granted ASM a non-exclusive, world-wide license to publish the Supplemental Material files. Please contact the corresponding author directly for reuse.
