# Supplementary material for: Lipidomics reveals the pro-viral roles of ceramides during fish nodavirus infection
Source: J Virol. 2026 Feb 23;100(3):e01991-25. doi: 10.1128/jvi.01991-25 (PMC13011388; doi:10.1128/jvi.01991-25)
Supplement: Supplemental material — Fig. S1 to Fig. S4; Tables S1 to S3. [file jvi.01991-25-s0001.docx]

**Supplemental figures and tables**

**
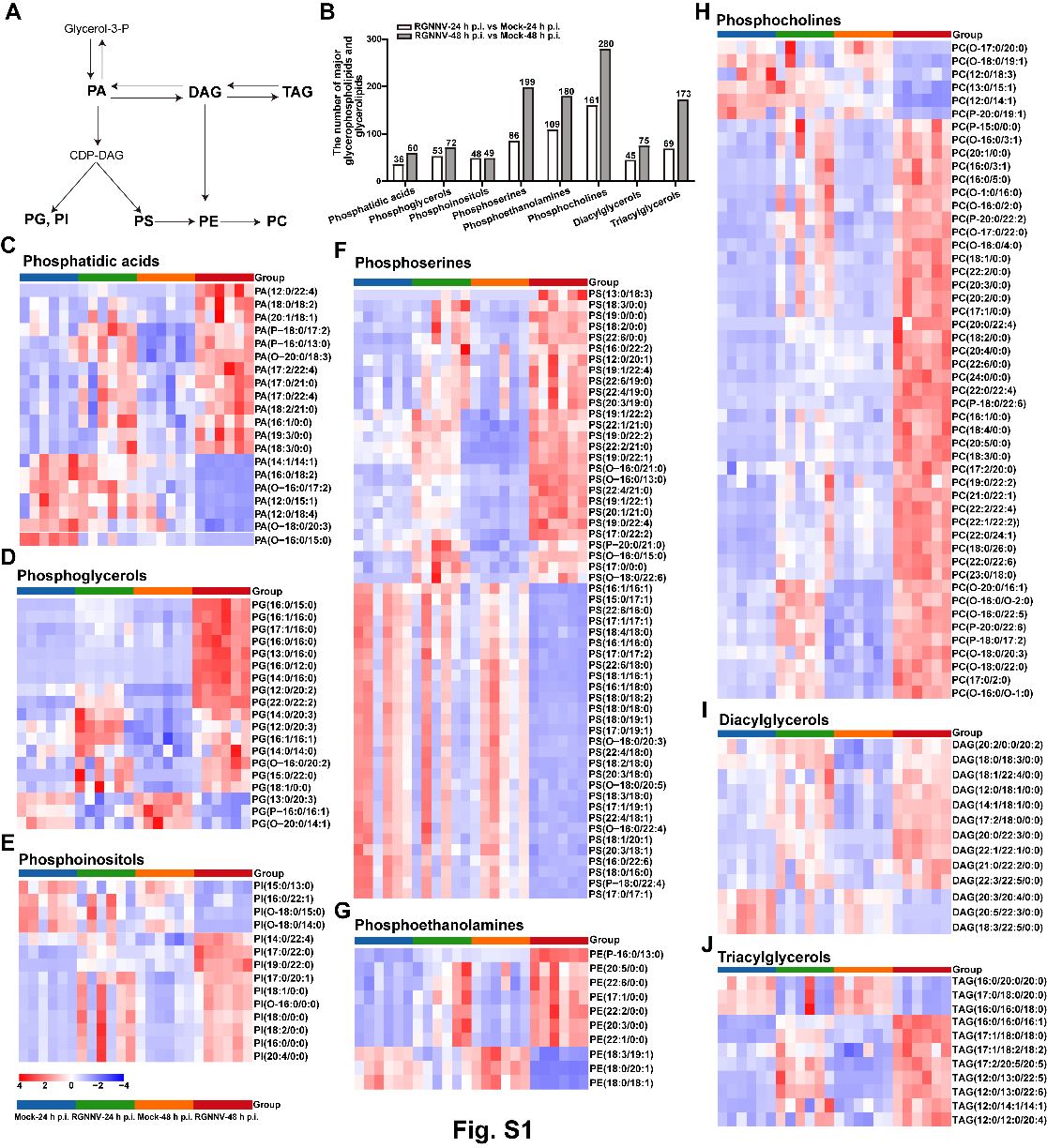
**

**Fig. S1 RGNNV infection altered lipid metabolites in GPs and GLs classes. (A)** A schematic diagram of the biosynthesis of GPs and GLs. Highlighted in bold are the GPs and GLs detected in this study. **(B)** The number of major altered lipid metabolites in GPs and GLs classes in RGNNV infected cells at 24 and 48 h p.i.. **(C-H)** The heatmap of representative GPs subclasses, including PA (**C**), PG (**D**), PI (**E**), PS (**F**), PE (**G**), and PC (**H**). **(I, J)** The heatmap of representative GLs, including DAG **(I)** and TAG **(J)**. Data were normalized by z-score and subjected to hierarchical clustering. The color gradient from blue (negative z-score, low expression) to red (positive z-score, high expression) indicated the degree of expression deviation. Abbreviations: glycerol-3-P, glycerol-3-phosphate; DAG, diradylglycerol; CDP-DAG, cytidine diphosphate diacylglycerol; TAG, triacylglycerol; PA, phosphatidic acid; PG, phosphoglycerol; PI, phosphoinositol; PC, phosphocholine; PE, phosphoethanolamine; PS, phosphoserine.

**
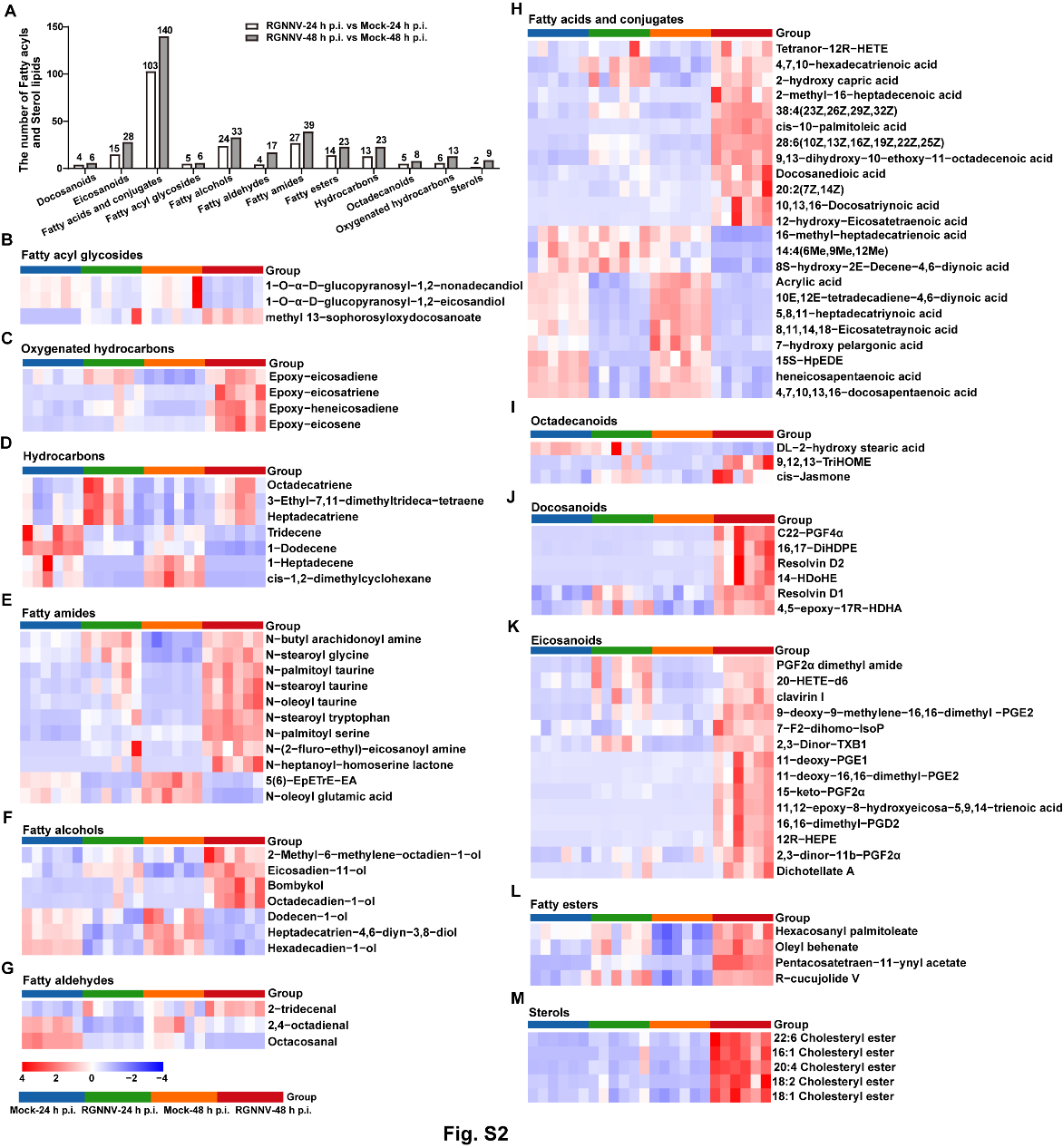
**

**Fig. S2 The levels of FAs and** **STs content were dynamically altered upon RGNNV infection *in vitro*. (A)** The number of changed lipid molecules in subclasses of FAs and STs. (**B-L**) The heatmap of representative FAs subclasses, including fatty acyl glycosides (**B**), oxygenated hydrocarbons (**C**), hydrocarbons (**D**), fatty amides (**E**), fatty alcohols (**F**), fatty aldehydes (**G**), fatty acids and conjugates (**H**), octadecanoids (**I**), docosanoids (**J**), eicosanoids (**K**), and fatty esters (**L**). **(M)** The levels of representative cholesterol esters in RGNNV infected cells. Data were normalized by z-score and subjected to hierarchical clustering. The color gradient from blue (negative z-score, low expression) to red (positive z-score, high expression) indicated the degree of expression deviation.


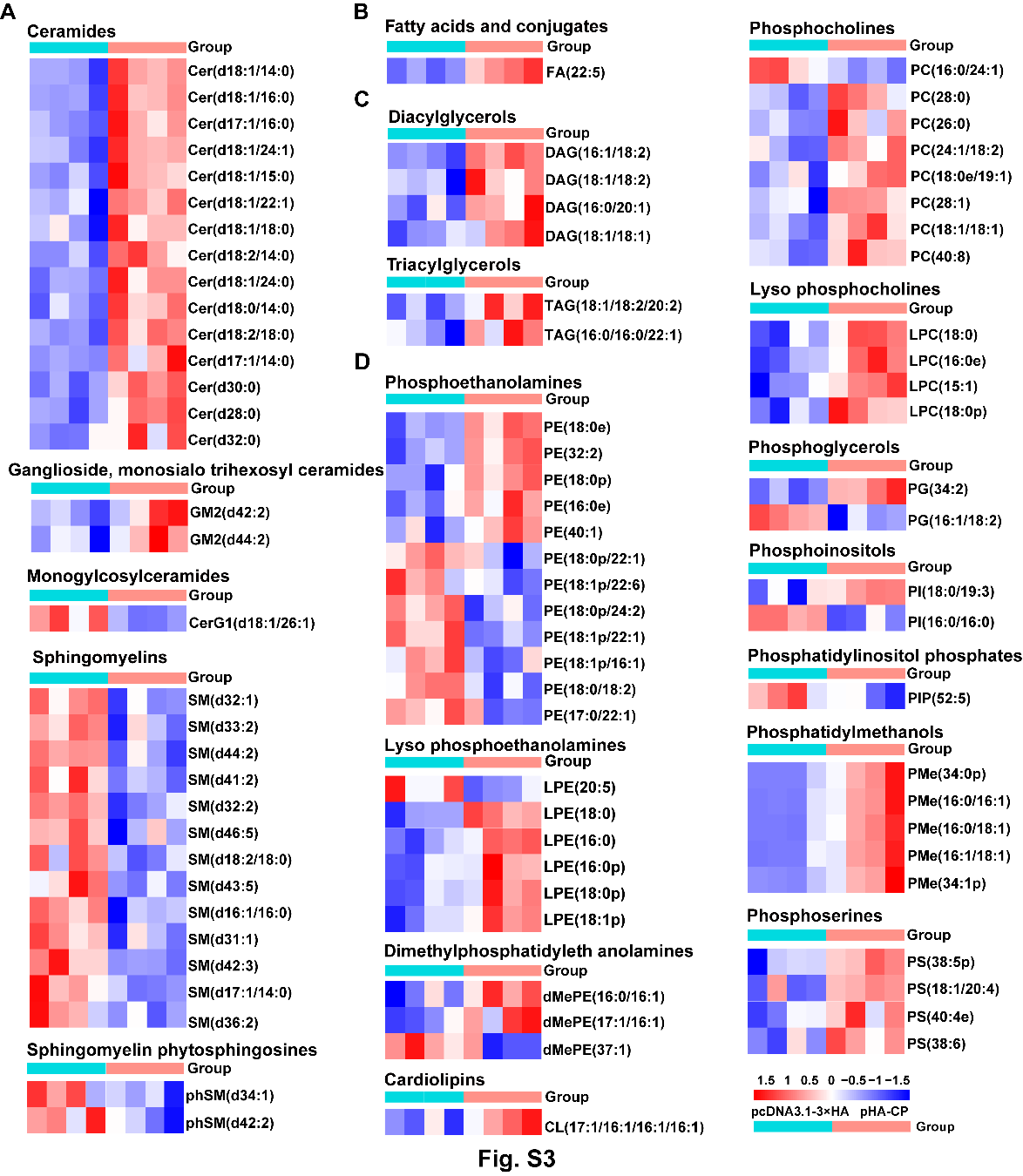


**Fig. S3 The level of identified lipids in pHA-CP-overexpressing cells. (A)** The heatmap of identified SPs subclasses, including ceramides (Cer), ganglioside, monosialo trihexosyl ceramides (GM), monogylcosylceramides (CerG), sphingomyelins (SM), and sphingomyelin phytosphingosines (phSM). **(B)** The heatmap of identified FAs subclasses. **(C)** The heatmap of identified GLs subclasses, including diacylglycerols (DAG), triacylglycerols (TAG). **(D)** The heatmap of representative GPs subclasses, including phosphatidylethanolamines (PE), lyso PE (LPE), dimethylphosphatidyleth anolamines (dMePE), cardiolipins (CL), phosphatidylcholines (PC), lyso PC (LPC), phosphatidylglycerols (PG), phosphatidylinositols (PI), phosphatidylinositol phosphates (PIP), phosphatidylmethanols (PMe), and phosphatidylserines (PS). Data were normalized by z-score and subjected to hierarchical clustering. The color gradient from blue (negative z-score, low expression) to red (positive z-score, high expression) indicated the degree of expression deviation.


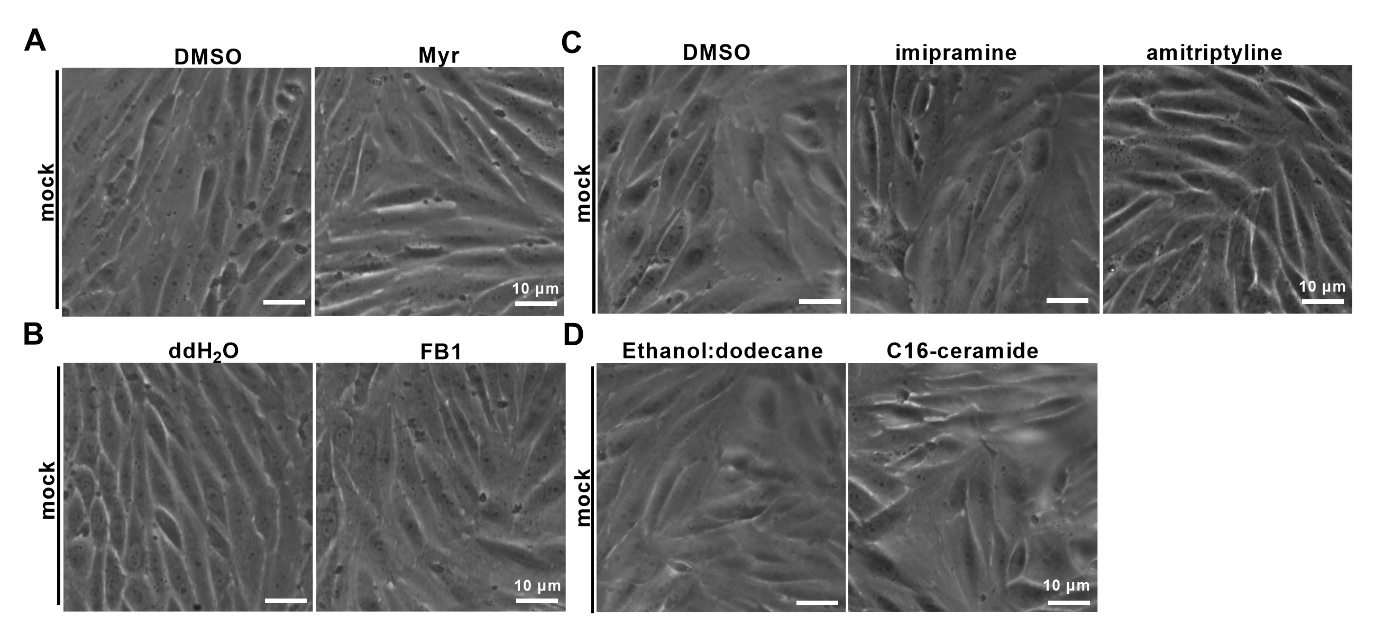


**Fig. S4 The morphology of uninfected cells treated with ceramide or inhibitors. (A-D)** GS cells were treated with 25 μM Myr **(A)**, 50 μM FB1 **(B)**, 10 μM imipramine, 10 μM amitriptyline **(C)**, or 60 μM C16-ceramide **(D)** for 48 h, then the cells morphology was observed and photographed under a microscope.

Table S1 The total number of the identified lipid classes and subclasses in RGNNV-infected cells.

| Classes | Subclasses | RGNNV-24 h p.i. vs Mock-24 h p.i. | | | RGNNV-48 h p.i. vs Mock-48 h p.i. | | |
| --- | --- | --- | --- | --- | --- | --- | --- |
|  |  | Total | Up | Down | Total | Up | Down |
| Sphingolipids (SPs) | Sphingoid bases (SB) | 8 | 2 | 6 | 14 | 3 | 11 |
|  | Amphoteric glycosphingolipids | 1 | 1 | 0 | 1 | 1 | 0 |
|  | Acidic glycosphingolipids (AGSL) | 11 | 11 | 0 | 8 | 7 | 1 |
|  | Neutral glycosphingolipids (NGSL) | 27 | 21 | 6 | 34 | 34 | 0 |
|  | Ceramides (Cer) | 13 | 5 | 8 | 34 | 28 | 6 |
|  | Sphingomyelins (SM) | 26 | 26 | 0 | 22 | 22 | 0 |
| Glycerophospholipids (GPs) | Phosphatidic acids (PA) | 36 | 18 | 18 | 60 | 25 | 35 |
|  | Phosphoglycerols (PG) | 53 | 28 | 25 | 72 | 36 | 36 |
|  | Phosphoinositols (PI) | 48 | 36 | 12 | 49 | 31 | 18 |
|  | Phosphoserines (PS) | 86 | 50 | 36 | 199 | 74 | 125 |
|  | Phosphoethanolamines (PE) | 109 | 32 | 77 | 180 | 72 | 108 |
|  | Phosphocholines (PC) | 161 | 105 | 56 | 280 | 160 | 120 |
|  | Oxidized glycerophospholipids | 7 | 3 | 4 | 7 | 3 | 4 |
|  | Phosphoglycerophosphates | 1 | 1 | 0 | 1 | 1 | 0 |
|  | Phosphoglycerophosphoglycerols | 2 | 0 | 2 | 2 | 0 | 2 |
|  | Phosphonocholines | 0 | 0 | 0 | 1 | 1 | 0 |
|  | Phosphonoethanolamines | 1 | 0 | 1 | 0 | 0 | 0 |
|  | Pyrophosphates | 1 | 1 | 0 | 1 | 1 | 0 |
|  | Other glycerophospholipids | 3 | 3 | 0 | 4 | 3 | 1 |
| Glycerolipids (GLs) | Monoacylglycerols (MAG) | 2 | 1 | 1 | 6 | 3 | 3 |
|  | Diacylglycerols (DAG) | 45 | 17 | 28 | 75 | 38 | 37 |
|  | Triacylglycerols (TAG) | 69 | 14 | 55 | 173 | 139 | 34 |
|  | Other glycerolipids | 1 | 0 | 1 | 2 | 1 | 1 |
|  | Glycosyldiradylglycerols | 3 | 2 | 1 | 6 | 4 | 2 |
| Fatty acyls (FAs) | Docosanoids | 4 | 4 | 0 | 6 | 6 | 0 |
|  | Eicosanoids | 15 | 9 | 6 | 28 | 17 | 11 |
|  | Fatty acids and conjugates | 103 | 39 | 64 | 140 | 66 | 74 |
|  | Fatty acyl glycosides | 5 | 1 | 4 | 6 | 2 | 4 |
|  | Fatty alcohols | 24 | 7 | 17 | 33 | 11 | 22 |
|  | Fatty aldehydes | 4 | 0 | 4 | 17 | 4 | 13 |
|  | Fatty amides | 27 | 18 | 9 | 39 | 20 | 19 |
|  | Fatty esters | 14 | 7 | 7 | 23 | 15 | 8 |
|  | Hydrocarbons | 13 | 7 | 6 | 23 | 8 | 15 |
|  | Octadecanoids | 5 | 3 | 2 | 8 | 3 | 5 |
|  | Oxygenated hydrocarbons | 6 | 5 | 1 | 13 | 8 | 5 |
|  | Other fatty acyls | 0 | 0 | 0 | 1 | 0 | 1 |
| Sterol lipids (STs) | Sterols | 2 | 1 | 1 | 9 | 7 | 2 |

Table S2 The total number of the identified lipid classes and subclasses in CP-overexpressing cells.

| Classes | Subclasses | pcDNA3.1-3×HA vs pHA-CP | | |
| --- | --- | --- | --- | --- |
|  |  | Total | Up | Down |
| Sphingolipids (SPs) | Ceramides (Cer) | 15 | 15 | 0 |
|  | Ganglioside, monosialo trihexosyl ceramides (GM) | 2 | 2 | 0 |
|  | Monogylcosylceramides (CerG) | 1 | 0 | 1 |
|  | Sphingomyelins (SM) | 13 | 0 | 13 |
|  | Sphingomyelin phytosphingosines (phSM) | 2 | 0 | 2 |
| Fatty acyls (FAs) | Fatty acids and conjugates (FA) | 1 | 1 | 0 |
| Glycerolipids (GLs) | Diacylglycerols (DAG) | 4 | 4 | 0 |
|  | Triacylglycerols (TAG) | 2 | 2 | 0 |
| Glycerophospholipids (GPs) | Phosphoethanolamines (PE) | 14 | 7 | 7 |
|  | Lyso phosphoethanolamines (LPE) | 7 | 6 | 1 |
|  | Dimethylphosphatidyleth anolamines (dMePE) | 3 | 2 | 1 |
|  | Cardiolipins (CL) | 1 | 1 | 0 |
|  | Phosphocholines (PC) | 12 | 9 | 3 |
|  | Lyso phosphocholines (LPC) | 10 | 10 | 0 |
|  | Phosphoglycerols (PG) | 2 | 1 | 1 |
|  | Phosphoinositols (PI) | 3 | 2 | 1 |
|  | Phosphatidylinositol phosphates (PIP) | 1 | 0 | 1 |
|  | Phosphatidylmethanols (PMe) | 6 | 6 | 0 |
|  | Phosphoserines (PS) | 6 | 5 | 1 |

Table S3. Primers used in this study.

| Primer names | Sequence (5’-3’) |
| --- | --- |
| β-actin-RT-F | TACGAGCTGCCTGACGGACA |
| β-actin-RT-R | GGCTGTGATCTCCTTCTGCA |
| Cers2a-RT-F | TGACCTGAAAGACCACGATG |
| Cers2a-RT-R | CTGGACCTGTCGCACTGAA |
| Cers6-RT-F | TATTTTGGCGTGGTTTTGG |
| Cers6-RT-R | CGTTGGGTTGTGCTTTCTG |
| SPTssA-RT-F | CCCTCGGTGACTGTTGGA |
| SPTssA-RT-R | CCAGGGAGTTGAACACGGT |
| SPTlc1-RT-F | TACAAGGCTTTACAGGGAACTC |
| SPTlc1-RT-R | GGTAACGAGCAAGGGTCAG |
| SPTlc2b-RT-F | GCCAACGGGAAGAAACC |
| SPTlc2b-RT-R | TCCTCAAACGACTCCACAAA |
| ASMase-RT-F | CGTAAATGAAGAGCGAGTAGCA |
| ASMase-RT-R | GACCTTTGGCAGGGTGATG |
| Atg5-RT-F | CCACTGAGGAGGGAGGCTT |
| Atg5-RT-R | CAGATGAAACAGGGCGAAA |
| Atg16L1-RT-F | GAACAGCCAACTCCTTCAGCACG |
| Atg16L1-RT-R | TCCTTTAGGCTCACAGCGACCAG |
| CP-RT-F | CAACTGACAACGATCACACCTTC |
| CP-RT-R | CAATCGAACACTCCAGCGACA |
| RdRp-RT-F | GTGTCCGGAGAGGTTAAGGATG |
| RdRp-RT-R | CTTGAATTGATCAACGGTGAACA |
| siRNA1-Cers2a | GGGCAGGGACUUUGAUCAUTT |
| siRNA2-Cers2a | GCGUAGCAUCGGAUGUCAATT |
| siRNA3-Cers2a | GGGCCGAUUGGAUUUGGUUTT |
| siRNA1-SPTlc2b | GGAAAGCUCCGCGAACAAATT |
| siRNA2-SPTlc2b | GCCUGUACAAGAAGCCCUUTT |
| siRNA3-SPTlc2b | GGACCUAGUGGAGAGAGUUTT |
